# Covariate Adjustment in Basket Trials Borrowing Information Across Subgroups

**DOI:** 10.1002/sim.70492

**Published:** 2026-03-19

**Authors:** Jiyang Ren, David S. Robertson, Haiyan Zheng

**Affiliations:** ^1^ Department of Statistics and Data Science Tsinghua University Beijing China; ^2^ MRC Biostatistics Unit University of Cambridge Cambridge UK; ^3^ Department of Mathematical Sciences University of Bath Bath UK

**Keywords:** ANCOVA, balancing covariates, Bayesian hierarchical model, borrowing strength, master protocols

## Abstract

Basket trials are an efficient approach to simultaneously evaluate a single therapy across multiple diseases where patients share a common molecular target. Bayesian hierarchical models (BHMs) are widely used to estimate the treatment effects while accounting for heterogeneity between patient subgroups within a basket trial. However, the use of analysis of covariance (ANCOVA) with treatment‐by‐covariate interaction terms, in this context of patient heterogeneity and small samples, has been largely unexplored, despite the widespread use of ANCOVA for improving estimation precision in traditional settings from a frequentist perspective. In this paper, we propose two covariate‐adjusted BHMs that incorporate ANCOVA into the data model to enhance the estimation precision in basket trials, wherein borrowing of information is permitted across subgroups to a certain extent. Specifically, both ANCOVA without treatment‐by‐covariate interaction terms and ANCOVA with interaction terms are explored in the analysis of basket trials. We perform a simulation study to demonstrate the advantages of covariate‐adjusted BHMs compared to unadjusted BHMs, as well as frequentist ANCOVA models. The BHMs are then retrospectively applied to the analysis of the MAJIC study, a randomized controlled basket trial involving two subtypes of blood cancer.

**Abbreviations**
ANCOVA: analysis of covariance
BHM: Bayesian hierarchical model

## 1 Introduction

In recent years, precision medicine has received considerable attention [1, 2, 3]. Particularly in oncology, treatments can be tailored to a patient's biomarkers (e.g., genetic mutations), rather than being solely guided by the tumor's tissue location. This tailored approach to treatment is referred to as ‘targeted therapy’, designed to inhibit the growth of cells that have a particular mutation [4, 5]. The evolution in anti‐cancer treatment has further led to the development of advanced designs and analysis strategies for clinical trials that involve a heterogeneous patient population.

One innovative method for evaluating targeted therapies is known as *basket trials*. Briefly, basket trials are a well‐used type of master protocol trial [6] designed to test a single targeted therapy across multiple diseases, whilst patients share the common molecular target [7, 8]. A pioneering example of basket trials is the phase II study evaluating vemurafenib in multiple nonmelanoma cancers with BRAF V600 mutations [9]. In this study, 122 cancer patients carrying BRAF V600 mutation were enrolled in six prespecified cancer cohorts and a seventh cohort with all other tumor types. Trial subsets (‘subtrials’ for short) are thus defined with respect to the disease subtypes, such as non‐small‐cell lung cancer, anaplastic thyroid cancer, etc.

Through basket trials, investigators aim not only to assess whether a drug is an effective biomarker‐targeted therapy but also to identify which tumor types are sensitive to the drug [10]. This motivates us to consider both overall efficacy and subtrial‐specific efficacy. Hobbs et al. [11] reviewed 13 pivotal basket trials in oncology and found that the primary analysis pooled data across histologies to evaluate an overall efficacy in seven studies, whereas the remaining six were designed for histology‐specific analyses. In practice, the choice of estimand is not universal and should be determined on a case‐by‐case basis. For example, not all basket trials are suited to targeting overall efficacy; an effective therapy may be deemed ineffective if one or more ineffective histologies are imbalanced in enrollment [10, 11]. Consequently, at the stage of planning basket trials, investigators generally need to consider whether the drug works in most or all subtrials, which leads to different strategies for the design and analysis. Therefore, to maximize applicability across basket trial settings, we focus on subtrial/histology‐specific treatment effects, and we also show in the Appendix that our method can be applied to estimate an overall efficacy in basket trials demonstrating homogeneous treatment effects.

Compared with conducting separate trials for various disease subtypes, basket trials are more efficient in terms of accelerated enrollment and simultaneous evaluation in one study under an overarching trial infrastructure. To acknowledge the commonality shared across subtrials (e.g., same genomic mutation), statistical modelling strategies that allow information borrowing for estimating the subtrial‐specific treatment effects are typically preferred over the approach of no borrowing (referred to as “stand‐alone analysis” below). However, one needs to be cautious about the degree of borrowing in the presence of high heterogeneity of treatment effects across disease subtypes. For instance, it has been observed that V600E BRAF‐mutant melanoma and hairy cell leukemia respond positively to BRAF inhibition, whereas colon tumors with the same BRAF mutation do not [7, 9, 12, 13, 14]. This poses challenges to perform information borrowing to an appropriate extent, which should ideally be determined by the level of heterogeneity.

The statistical literature on carefully handling heterogeneity in the analysis of basket trials is extensive, in both frequentist and Bayesian frameworks. Early proposals mainly concern stand‐alone analyses, that is, to estimate the treatment effects independently for each disease type [15]. While heterogeneity can be fully accounted for, this approach is limited by the small sample size of subtrials and inevitably leads to low‐powered tests. More recent proposals include the so‐called “test‐then‐pool” solution [10, 16, 17, 18], which proceeds with pooling only subtrials that have passed a homogeneity test. Such frameworks, nevertheless, can be prone to the risk of misclassification, and pooling heterogeneous subtrials can lead to biased estimation.

In contrast, Bayesian hierarchical models (BHMs) are advantageous, well‐studied, and widely utilized for information borrowing. In particular, BHMs permit information borrowing through the population parameters; specifically, the population variance captures the between‐subtrial heterogeneity. Thall et al. [19] proposed using BHMs to analyze Phase II clinical trials involving multiple diseases. Berry et al. [20] applied this approach to basket trials based on the assumption of exchangeability, which suggests that there is no prior evidence indicating a superior treatment effect in certain tumor types compared to others. Some research has relaxed this assumption by allowing the possibility of non‐exchangeability [21], employing mixtures of several BHMs [22, 23, 24], and utilizing clustering techniques [25, 26, 27]. Additional extensions—such as calibrated BHM [28], BHM with correlated priors [29], and BHM with commensurate priors [30]—have further generalized BHMs for information borrowing. To address the possibility that the model can be misspecified, Bayesian model averaging methods have been developed [31, 32, 33, 34]. Candidate models that capture the underlying heterogeneity among tumor types are associated with weights for averaging. To the best of our knowledge, however, little attention has been given to explicitly incorporating covariate information that may contribute to the heterogeneity of treatment effects besides tumor (sub)types (the subgroups indicator).

Covariate adjustment using generalized linear models is widely accepted and guided by industry standards as outlined in ICH E9 and the FDA guidance [35, 36], although these guidelines do not specifically address the application in Bayesian frameworks. Covariates typically include both predictive and prognostic factors. Prognostic factors can only affect outcomes, while predictive factors can affect both the outcomes and treatment effects. The FDA guidance notes that “incorporating prognostic baseline factors in the analysis of clinical trial data can result in a more efficient use of data to demonstrate and quantify the effects of treatment with minimal impact on bias or the Type I error rate”, which emphasizes the benefit of covariate adjustment on estimation precision (the variance of the estimator, also known as estimation efficiency). Among methods for covariate adjustment in randomized clinical trials, the analysis of covariance (ANCOVA) without or with treatment‐by‐covariate interaction terms (referred to below as “ANCOVA I” and “ANCOVA II”, respectively) has been extensively studied [37, 38, 39, 40]. The advantages of ANCOVA I and II in studies with simple randomization have been demonstrated [37]. In our review of the literature, ANCOVA I (without treatment‐by‐covariate interaction terms) is more frequently applied than ANCOVA II (with interaction terms) for clinical trials drawing inferences in a Bayesian framework [30, 41, 42, 43, 44]. This has motivated us to revisit this topic. Specifically, it remains unclear if excluding treatment‐by‐covariate interaction terms would result in a loss in precision under BHMs, as was demonstrated from the frequentist perspective [37, 39, 45].

The rest of the paper is organized as follows. Section 2 introduces three data models (i.e., one unadjusted model and two covariate‐adjusted models), followed by a common parameter model, to form BHMs for borrowing of information in basket trials. Section 3 describes a simulation study to evaluate the performance of different methods, including BHMs and frequentist ANCOVA models. Section 4 applies the methods to hypothetical data simulated based on the MAJIC study. Section 5 concludes the paper with a discussion about future research avenues.

## 2 Methods

Consider a basket trial with K subtrials. Let $\theta_{k}$ denote the average treatment effect and $n_{k}$ the sample size specific to each subtrial k=1,…,K. Throughout this paper, we consider a randomized controlled setting where the $n_{k}$ patients are randomly assigned between a new treatment and control within each subtrial, with $n_{1k}$ patients in the treatment group and $n_{0k}$ patients in the control group. That is, $n_{k}=n_{1k}+n_{0k}$. The treatment arms, as well as the randomization ratio (denoted by $n_{1k}:n_{0k}$), are kept the same across subtrials. For patient $i=1,\ldots ,n_{k}$ within subtrial k, let $T_{ik}$ denote the treatment assignment indicator, with $T_{ik}=1$ representing the treatment and $T_{ik}=0$ representing the control. Let $Y_{ik}$ denote the continuous outcome of interest and $X_{ik}$ the pretreatment (baseline) covariates.

### 2.1 Data Models: Unadjusted, ANCOVA I and ANCOVA II

To start with, we describe an *unadjusted* data model that does not consider the covariate information. Assume the relationship between outcome, denoted by $Y_{ik}$, and the treatment, denoted by $T_{ik}$, can be characterized by a normal linear regression model

(1) $$E(Y_{ik}|T_{ik})=\alpha_{k}+\theta_{k}T_{ik},$$

where $\alpha_{k}$ is the intercept and $\theta_{k}$ is the treatment effect parameter of subtrial k. Here, we consider a continuous outcome, but this modelling assumption is not restrictive in the sense that other data types can be covered through the use of a generalized linear model. With an unadjusted analysis strategy,

$$E(Y_{ik}|T_{ik}=1)=\alpha_{k}+\theta_{k},\;E(Y_{ik}|T_{ik}=0)=\alpha_{k}.$$

for each subtrial k=1,…,K.

As noted, baseline covariates can influence the outcome and the treatment effect. Adjusting for the imbalance of covariates in the analysis of randomized trials potentially decreases the asymptotic variance of treatment effect estimators. Basket trials consist of K subtrials, with each implementing a simple randomization mechanism, that is, complete randomization at a fixed ratio. It is hence intuitively appealing to extend the covariate adjustment methods, devised for a homogeneous population with a simple randomization mechanism, to basket trials to improve the estimation efficiency, and accordingly to reduce the asymptotic variance of the estimator.

ANCOVA has been well‐studied for simple randomization under both the super‐population framework and the finite‐population framework [37, 39, 45]. The former concerns the randomness of random sampling and random treatment assignment, while the latter considers random treatment assignment alone. In brief, ANCOVA is divided into two cases: ANCOVA I models regress the outcome on the treatment indicator and covariates to adjust the effect of covariates on the outcome; ANCOVA II models build on the ANCOVA I model to further include interaction terms of the treatment and the covariates, which further adjust the effect of covariates. In a homogeneous population with a simple randomization mechanism, Yang and Tsiatis [37] compared ANCOVA I, ANCOVA II, and the unadjusted method, concluding that:

1. ANCOVA II is the most efficient among these three methods;
2. Under equal allocation, ANCOVA I is as efficient as ANCOVA II;
3. ANCOVA I cannot guarantee efficiency gains compared with the unadjusted method.

For basket trials that involve several subpopulations, we consider two data models extended from ANCOVA I and ANCOVA II, respectively. Note that $\theta_{k}$ refers to a subtrial‐specific treatment effect. The purpose of covariate adjustment is to adjust for predictive factors within each subtrial, other than for tumor types that define the subtrials.

We first consider the ANCOVA I model, assuming that (prognostic) covariates can affect outcomes while not affecting treatment effects:

(2) $$E(Y_{ik}|T_{ik},X_{ik})=\alpha_{k}+\theta_{k}T_{ik}+\beta_{k}X_{ik},$$

where $\alpha_{k}$ is the intercept, $\theta_{k}$ is the treatment effect, and $\beta_{k}$ is the effect of covariates on the outcome of subtrial k. For each subtrial k, we have

$$\begin{array}{cc} E(Y_{ik}|T_{ik}=1) & =\alpha_{k}+\theta_{k}+\beta_{k}E(X_{ik}),\\ E(Y_{ik}|T_{ik}=0) & =\alpha_{k}+\beta_{k}E(X_{ik}). \end{array}$$

For the ANCOVA II model, we assume that the (predictive) covariates can affect both outcomes and treatment effects:

(3) $$E(Y_{ik}|T_{ik},X_{ik})=\alpha_{k}+\theta_{k}T_{ik}+\beta_{k}X_{ik}+\gamma_{k}T_{ik}X_{ik},$$

where $\alpha_{k}$ is the intercept, $\theta_{k}$ is the treatment effect parameter, $\beta_{k}$ is the effect of covariates on the outcome, and $\gamma_{k}$ is the effect of covariates on the treatment effect of subtrial k. For each subtrial k, we have

$$\begin{array}{cc} E(Y_{ik}|T_{ik}=1) & =\alpha_{k}+\theta_{k}+(\beta_{k}+\gamma_{k})E(X_{ik}),\\ E(Y_{ik}|T_{ik}=0) & =\alpha_{k}+\beta_{k}E(X_{ik}), \end{array}$$

$$\;E(Y_{ik}|T_{ik}=1)-E(Y_{ik}|T_{ik}=0)=\theta_{k}+\gamma_{k}E(X_{ik}).$$

Note that we need to assume the subtrial‐specific means of covariates equal 0, that is, $E(X_{ik})={\bar{X}}_{k}=0$ for each k. If this is not met by default, one can use $X_{ik}-{\bar{X}}_{k}$ for standardization to center around 0 in the data analysis. Otherwise, $\theta_{k}$ is no longer the subtrial‐specific average treatment effect.

### 2.2 Parameter Model: Borrowing of Information Across Subtrials

Following Thall et al. [19], we fit a BHM to estimate the subtrial‐specific treatment effects, $\theta =(\theta_{1},\ldots ,\theta_{K})$, by including a parameter model as follows:

(4) $$\begin{array}{cc} \theta_{k}|\mu_{\theta },\sigma_{\theta } & ∼N(\mu_{\theta },\sigma_{\theta }^{2}),\;\text{for}\;k=1,\ldots ,K\\ \mu_{\theta } & ∼N(\mu ,\sigma^{2}),\\ \sigma_{\theta } & ∼HT(\sigma_{HT},d), \end{array}$$

where $\mu_{\theta }$ and $\sigma_{\theta }^{2}$ are the unknown population mean and variance. Specifically, $\mu_{\theta }$ influences the locations of the subtrial‐specific treatment effects, and $\sigma_{\theta }$ represents the level of heterogeneity between subtrials. Borrowing of information is permitted through an underlying distribution, from which values for $\sigma_{\theta }$ are drawn. Here, we have placed a $HT(\sigma_{HT},d)$ prior on $\sigma_{\theta }$, which denotes a half‐t distribution. That is, a generalized t distribution with a location parameter of 0, a scale parameter of $\sigma_{HT}$, and d degrees of freedom, truncated to take positive numbers only. The parameter $\sigma_{HT}$ controls the degree of borrowing: the higher the value of $\sigma_{HT}$, the less information is borrowed between subtrials. For implementation, the hyperparameters, $\mu ,\;\sigma ,\;\sigma_{HT},\;d$, are prespecified. Alternatively, an inverse‐Gamma or Uniform prior can be specified for $\sigma_{\theta }$. Here, we choose a heavily‐tailed half‐t prior, as it is less sensitive to the hyperparameters, based on the simulation results in Cunanan et al. [46]. We refer the interested reader to Gelman [47] and Cunanan et al. [46] for general recommendations on priors for variance parameters.

This parameter model (4) coupled with each of the three data models outlined in Section 2.1, results in three hierarchical models to estimate the substrial‐specific treatment effects θ. To complete the BHMs, we specify priors for the other parameters. Specifically, $\alpha_{k},\;\beta_{k},\;\gamma_{k}$ each follow a normal prior, and $\sigma_{y}^{2}$, the variance of the outcome, follows an inverse‐Gamma prior. Hereafter, we refer to these three hierarchical Bayesian ANCOVA methods as unadjusted BHM (BHM), adjusted BHM without interaction (adjBHM), and adjusted BHM with interaction (intBHM), respectively. A recap of the three BHMs considered is provided in Table 1. We fit the BHMs using Markov chain Monte Carlo (MCMC) to draw samples from the posterior distributions, and then estimate the subtrial‐specific treatment effects θ.

**TABLE 1:** Summary of the three Bayesian hierarchical models considered.

	Data model	Parameter model
BHM	$E(Y_{ik}|T_{ik})=\alpha_{k}+\theta_{k}T_{ik}$	Equation (4)
adjBHM	$E(Y_{ik}|T_{ik},X_{ik})=\alpha_{k}+\theta_{k}T_{ik}+\beta_{k}X_{ik}$	
intBHM	$E(Y_{ik}|T_{ik},X_{ik})=\alpha_{k}+\theta_{k}T_{ik}+\beta_{k}X_{ik}+\gamma_{k}T_{ik}X_{ik}$	

## 3 Simulation Study

In this section, we conduct a simulation study to evaluate the performance of unadjusted BHM, adjBHM, and intBHM, and two classes of frequentist methods, stratified AN(C)OVAs and random‐effect AN(C)OVAs, in analyzing basket trials with K=4 subtrials.

### 3.1 Design of the Simulation Study

#### 3.1.1 Aims

We aim to compare the performance of covariate‐adjusted with unadjusted BHM as well as Bayesian with frequentist methods, considering the impact of correctly specified or mis‐specified data‐generating models, different sample sizes, and different treatment allocation ratios.

#### 3.1.2 Data‐Generating Mechanisms

We consider four data‐generating mechanisms. For all, we consider basket trials with K=4 subtrials. The sample size ratio of different subtrials is $n_{1}:n_{2}:n_{3}:n_{4}=2:3:4:5$. A range of different sample sizes are set for the numerical evaluation so that the total sample size, $n=\sum_{k=1}^{K}n_{k}$, ranges from 56 to 560 in increments of 56. The ratio 2:3:4:5 is motivated by the practical consideration that basket trials often have a varying number of patients involved. We let the heterogenous treatment effects θ=(0,0,1,1) (with a homogeneous scenario with θ=(1,1,1,1) analyzed in Appendix B), and one‐dimensional pretreatment covariate, $X_{ik}\overset{i.i.d.}{∼}N(0,1),\forall k=1,\ldots ,4$.

We generate the outcomes from using the following three models:

1. $Y_{ik}=1+\theta_{k}T_{ik}-2X_{ik}+4T_{ik}X_{ik}+\epsilon_{ik}$,
2. $Y_{ik}=1+\theta_{k}T_{ik}-2X_{ik}+\epsilon_{ik}$,
3. $Y_{ik}=1+\theta_{k}T_{ik}-2X_{ik}+T_{ik}X_{ik}^{3}+\epsilon_{ik}$,

where $\epsilon_{ik}$ are i.i.d. standard normal random noise. Scenario (1) is correctly specified for ANCOVA II but mis‐specified for ANCOVA I, scenario (2) is correctly specified for ANCOVA I and the degenerate ANCOVA II with $\gamma_{k}=0$, even if ANCOVA II requires estimating one more parameter $\gamma_{k}$, and scenario (3) is mis‐specified for all of the methods. We consider the most commonly used randomization ratio of 1:1 for each of the K subtrials, that is, a 1:1 allocation of treatment:control; equivalently, $n_{1k}/n_{k}=0.5$ where $n_{1k}$ denotes the number of patients allocated to the treatment. In order to study whether unequal allocation ratios would affect the consistency of the conclusions, in scenario (1), we also consider a 3:1 allocation of treatment:control, that is, $n_{1k}/n_{k}=0.75$ for all the subtrials.

#### 3.1.3 Estimands

Our estimands are subtrial‐specific treatment effects $\theta_{k}$.

#### 3.1.4 Methods

Each of the three BHMs three stratified AN(C)OVA models, and three random‐effect AN(C)OVA models, is fitted to the simulated trial data to compare the performance of different methods. The specification of different models and their abbreviations are summarized in Table 2.

**TABLE 2:** Different models for performance comparison.

Abbreviation	Model
BHM	Unadjusted BHM with a HT(2,5) prior on $\sigma_{\theta }$
adjBHM	Covariate‐adjusted BHM without interaction term (ANCOVA I) with a HT(2,5) prior on $\sigma_{\theta }$
intBHM	Covariate‐adjusted BHM with interaction term (ANCOVA II) with a HT(2,5) prior on $\sigma_{\theta }$
BHM2	Unadjusted BHM with a HT(100,5) weak prior on $\sigma_{\theta }$
adjBHM2	Covariate‐adjusted BHM without interaction term (ANCOVA I) with a HT(100,5) weak prior on $\sigma_{\theta }$
intBHM2	Covariate‐adjusted BHM with interaction term (ANCOVA II) with a HT(100,5) weak prior on $\sigma_{\theta }$
sANOVA	Unadjusted stratified ANOVA
sANCOVAI	Covariate‐adjusted stratified ANCOVA I
sANCOVAII	Covariate‐adjusted stratified ANCOVA II
reANOVA	Unadjusted random‐effect ANOVA with random slope of $T_{ik}$
reANCOVAI	Covariate‐adjusted random‐effect ANCOVA I with random slope of $T_{ik}$
reANCOVAII	Covariate‐adjusted random‐effect ANCOVA II with random slope of $T_{ik}$

As noted earlier, we use cluster‐centered covariates $X_{ik}-{\bar{X}}_{k}$ to perform covariate adjustment. For Bayesian methods, we set an inverse−Gamma(2,20) prior on the variance of the outcome, a N(0,100) prior on $\mu_{\theta }$, $\alpha_{k}$, $\beta_{k}$, $\gamma_{k}$, and a HT(2,5) or a HT(100,5) weak prior on $\sigma_{\theta }$. For Bayesian methods, we apply MCMC using a burn‐in of 2000 with a chain of length 10 000 to sample the posteriors and use posterior means with 95% credible intervals symmetric about the posterior mean, to estimate $\theta_{k}$. For stratified AN(C)OVA models, we fit linear regression data models in Section 2.1 separately within each subtrial to estimate subtrial‐specific treatment effects. For random‐effect AN(C)OVA models, in the random effect part, we include random intercept and random slope of $T_{ik}$. In the fixed effect part, the random‐effect ANOVA model (reANOVA) includes treatment only, random‐effect ANCOVA I model (reANCOVAI) additionally includes covariates‐by‐subtrial interaction term, and the random‐effect ANCOVA II model (reANCOVAII) further includes treatment‐by‐covariates‐by‐subtrial interaction term.

#### 3.1.5 Performance Measures

We simulate B=1000 replicates of basket trials following the configuration above to generate the trial data. We compare the performance of these models in terms of the following metrics:

- bias, defined as the absolute difference between the mean of the empirical distribution of $\theta_{k}$ estimates and the true value used for simulating the data,
- standard deviation (SD), defined as the standard deviation of the estimates for $\theta_{k}$,
- root mean squared error (RMSE) of the estimates for $\theta_{k}$,
- RMSE reduction with respect to the unadjusted BHM,
- marginal rejection rate, defined as the proportion of simulated (sub)trials rejecting the null hypothesis $H_{0k}:\theta_{k}=0$ for each k, that is, where the posterior credible interval or confidence interval does not include the null value,
- family‐wise type I error rate, defined as the empirical probability of rejecting $H_{01}$ or $H_{02}$,
- disjunctive power, defined as the proportion of simulated (sub)trials rejecting $H_{03}$ or $H_{04}$.

The definitions, estimates, and Monte Carlo standard errors (MCSEs) for various performance metrics, such as bias, SD, RMSE, RMSE reduction for method 2 versus method 1, and marginal rejection rate, are provided in Table 3. To further clarify whether the observed differences in simulation performance stem from methodological differences or simulation error, we also report the MCSE for each of the performance metrics.

**TABLE 3:** Definitions, estimates and Monte Carlo standard errors of different performance metrics.

Metrics	Definition	Estimate	MCSE of estimate
Bias	$E({\hat{\theta }}_{k})-\theta_{k}$	$\sum_{b=1}^{B}({\hat{\theta }}_{k}^{b}-\theta_{k})/B$	$\sqrt{\sum_{b=1}^{B}{\left({\hat{\theta }}_{k}^{b}-{\bar{\hat{\theta }}}_{k}\right)}^{2}/B(B-1)}$
SD	$\sqrt{Var({\hat{\theta }}_{k})}$	$\sqrt{\sum_{b=1}^{B}{\left({\hat{\theta }}_{k}^{b}-{\bar{\hat{\theta }}}_{k}\right)}^{2}/(B-1)}$	${\hat{\text{SD}}}_{k}/\sqrt{2(B-1)}$
RMSE	$\sqrt{E[{\left({\hat{\theta }}_{k}-\theta_{k}\right)}^{2}]}$	$\sqrt{\sum_{b=1}^{B}{\left({\hat{\theta }}_{k}^{b}-\theta_{k}\right)}^{2}/B}$	$\frac{1}{2}\sqrt{\sum_{b=1}^{B}{\left[{\left({\hat{\theta }}_{k}^{b}-\theta_{k}\right)}^{2}-{\hat{\text{MSE}}}_{k}\right]}^{2}/\{B(B-1){\hat{\text{MSE}}}_{k}}\}$
RMSE reduction	$(1-{\text{RMSE}}_{k2}/{\text{RMSE}}_{k1})\times 100$	$(1-{\hat{\text{RMSE}}}_{k2}/\hat{{\text{RMSE}}_{k1}})\times 100$	$\hat{\text{MCSE}}\{{\text{MSE}}_{k2}/{\text{MSE}}_{k1}\}{\hat{\text{RMSE}}}_{k1}/{\hat{\text{RMSE}}}_{k2}\times 50$
Rejection rate	$Pr(0\notin [L_{k},U_{k}])$	$\sum_{b=1}^{B}I\{0\notin [{\hat{L}}_{k}^{b},{Û}_{k}^{b}]\}/B$	$\sqrt{{\hat{rate}}_{k}(1-{\hat{rate}}_{k})/B}$

*Note:* NB: ${\bar{\hat{\theta }}}_{k}$ denote the sample mean of B=1000 replicated $\theta_{k}$ estimates. ${\text{MSE}}_{k1}$ and ${\text{MSE}}_{k2}$ denote the mean squared error of the estimates for $\theta_{k}$ (${\text{MSE}}_{k}$) using method 1 and method 2, respectively. The MCSE of ${\text{MSE}}_{k}$ can be estimated as $\hat{\text{MCSE}}\{{\text{MSE}}_{k}\}=\sqrt{\sum_{b=1}^{B}{\left[{\left({\hat{\theta }}_{k}^{b}-\theta_{k}\right)}^{2}-{\hat{\text{MSE}}}_{k}\right]}^{2}/B(B-1)}$, and the MCSE of ${\text{MSE}}_{k2}/{\text{MSE}}_{k1}$ can be estimated as $\hat{\text{MCSE}}\{{\text{MSE}}_{k2}/{\text{MSE}}_{k1}\}=\hat{\text{MCSE}}\{{\text{MSE}}_{k2}\}/{\left({\hat{\text{MSE}}}_{k1}\right)}^{2}+{\left({\hat{\text{MSE}}}_{k2}\right)}^{2}\hat{\text{MCSE}}\{{\text{MSE}}_{k1}\}/{\left({\hat{\text{MSE}}}_{k1}\right)}^{4}-2{\hat{\text{MSE}}}_{k2}\hat{Cov}\{{\text{MSE}}_{k1},{\text{MSE}}_{k2}\}/{\left({\hat{\text{MSE}}}_{k1}\right)}^{3}$. ${\hat{L}}_{k}$ and ${Û}_{k}$ denote the lower and upper bounds of the 95% posterior credible intervals or confidence intervals of $\theta_{k}$, respectively. The superscript b refers to the estimates of the bth simulation replicate.

### 3.2 Exploration and Visualization of Results

#### 3.2.1 Results of Scenario (1) With $n_{1k}/n_{k}=0.75$

**FIGURE 1:**
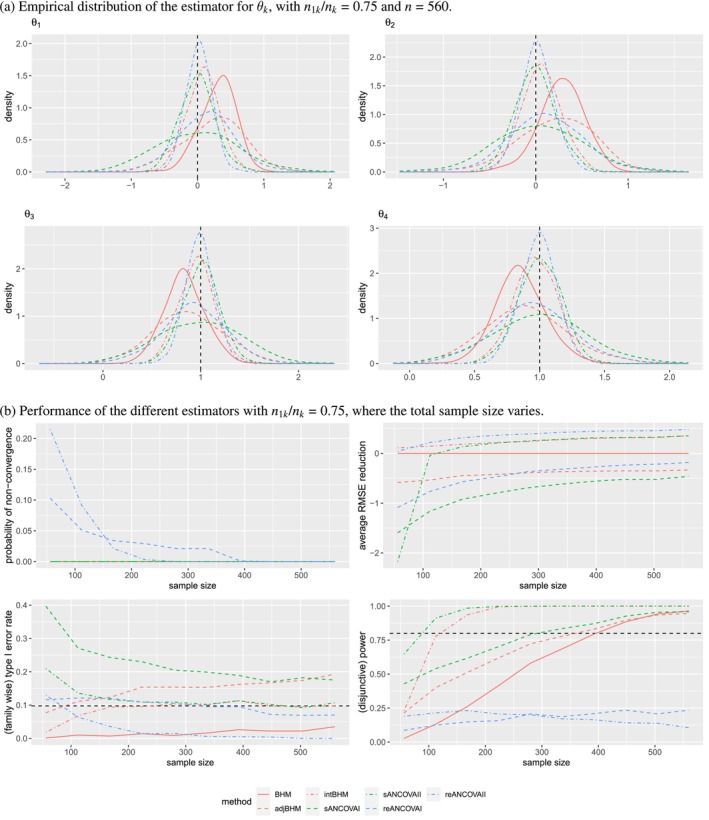
Simulation results of the different estimators under scenario (1) with $n_{1k}/n_{k}=0.75$. The dashed horizontal lines in Panel (b) represent the 9.75% level of (family‐wise) type I error rate and 80% (disjunctive) power, respectively. Only seven methods (BHM, adjBHM, intBHM, sANCOVAI, sANCOVAII, reANCOVAI, reANCOVAII) are included in this figure.

Results of scenario (1) with $n_{1k}/n_{k}=0.75$ are shown in Figure 1 and Table 4. Note that only seven methods (BHM, adjBHM, intBHM, sANCOVAI, sANCOVAII, reANCOVAI, reANCOVAII) are included in the figure to maintain differentiation between the curves and the readability of the plots. From Figure 1a and Table 4, we observe that for large sample sizes, in terms of the bias of the estimators of $\theta_{k}$, only stratified AN(C)OVAs have negligible biases in accordance with their theoretical unbiasedness. Except for stratified AN(C)OVAs, all the BHMs and random‐effect AN(C)OVAs have obvious bias; however, these biases can be almost entirely eliminated by using covariate adjustment with an interaction term because of correct specification. For BHMs, a weaker prior of $\sigma_{\theta }$ in this heterogenous scenario can help to reduce bias. Second, in terms of SD and RMSE, compared with unadjusted methods, all the covariate adjustments with the interaction term perform better, whereas covariate adjustment without the interaction term performs worse. For example, compared with unadjusted BHM, intBHM reduces the RMSE by 31%–41%, while adjBHM increases the RMSE by 23%–44%. Third, in terms of the marginal rejection rate, unadjusted methods and covariate‐adjusted methods with an interaction term can control the marginal type I error rate at 5% while covariate‐adjusted methods without an interaction term do not. For subtrials k=3 and 4, intBHM has the largest marginal power, whereas random‐effect methods lose power because of the violation of the normality assumption given the small number of subtrials.

**TABLE 4:** Simulation performance of the different estimators of $\theta_{k}$ yielded by each BHM and frequentist method with Monte Carlo simulation error in parentheses, for k=1,…,4, under scenario (1) with $n_{1k}/n_{k}=0.75$ and n=560.

		Bias	SD	RMSE	RMSE reduction (%)	Rejection rate
	BHM	0.308 (0.008)	0.265 (0.006)	0.406 (0.007)	—	0.018 (0.004)
	adjBHM	0.226 (0.015)	0.473 (0.011)	0.524 (0.011)	−28.961 (1.82)	0.105 (0.01)
	intBHM	0.082 (0.008)	0.244 (0.005)	0.258 (0.006)	36.572 (1.308)	0.047 (0.007)
	BHM2	0.262 (0.009)	0.289 (0.006)	0.39 (0.007)	4.13 (0.363)	0.011 (0.003)
	adjBHM2	0.187 (0.016)	0.502 (0.011)	0.536 (0.012)	−31.819 (2.128)	0.099 (0.009)
	intBHM2	0.068 (0.008)	0.249 (0.006)	0.258 (0.006)	36.538 (1.337)	0.045 (0.007)
$\theta_{1}=0$	sANOVA	0 (0.012)	0.38 (0.008)	0.38 (0.009)	6.561 (1.922)	0.006 (0.002)
	sANCOVAI	0.004 (0.019)	0.607 (0.014)	0.607 (0.014)	−49.406 (3.271)	0.101 (0.01)
	sANCOVAII	−0.004 (0.008)	0.261 (0.006)	0.261 (0.006)	35.689 (1.581)	0.055 (0.007)
	reANOVA	0.151 (0.007)	0.233 (0.005)	0.278 (0.006)	31.672 (1.119)	0.016 (0.004)
	reANCOVAI	0.128 (0.014)	0.437 (0.01)	0.455 (0.012)	−12.041 (2.394)	0.066 (0.008)
	reANCOVAII	0.021 (0.006)	0.191 (0.004)	0.192 (0.005)	52.739 (1.122)	0 (0)
	BHM	0.282 (0.008)	0.239 (0.005)	0.369 (0.006)	—	0.02 (0.004)
	adjBHM	0.216 (0.013)	0.4 (0.009)	0.455 (0.009)	−23.177 (1.47)	0.111 (0.01)
	intBHM	0.061 (0.007)	0.21 (0.005)	0.218 (0.005)	40.926 (1.213)	0.054 (0.007)
	BHM2	0.24 (0.008)	0.254 (0.006)	0.349 (0.006)	5.439 (0.313)	0.015 (0.004)
	adjBHM2	0.184 (0.013)	0.417 (0.009)	0.456 (0.009)	−23.441 (1.688)	0.1 (0.009)
	intBHM2	0.05 (0.007)	0.212 (0.005)	0.218 (0.005)	41.006 (1.244)	0.055 (0.007)
$\theta_{2}=0$	sANOVA	0.019 (0.009)	0.297 (0.007)	0.297 (0.007)	19.533 (1.603)	0.002 (0.001)
	sANCOVAI	0.04 (0.015)	0.47 (0.011)	0.471 (0.011)	−27.673 (2.646)	0.081 (0.009)
	sANCOVAII	−0.003 (0.007)	0.215 (0.005)	0.215 (0.005)	41.782 (1.404)	0.056 (0.007)
	reANOVA	0.12 (0.007)	0.224 (0.005)	0.254 (0.006)	31.088 (1.052)	0.014 (0.004)
	reANCOVAI	0.11 (0.012)	0.384 (0.009)	0.399 (0.009)	−8.17 (1.896)	0.059 (0.007)
	reANCOVAII	0.015 (0.006)	0.176 (0.004)	0.177 (0.004)	52.1 (1.166)	0 (0)
	BHM	−0.165 (0.006)	0.204 (0.005)	0.263 (0.005)	—	0.813 (0.012)
	adjBHM	−0.126 (0.011)	0.352 (0.008)	0.374 (0.008)	−42.426 (1.829)	0.766 (0.013)
	intBHM	−0.041 (0.006)	0.176 (0.004)	0.18 (0.004)	31.401 (1.536)	1 (0)
	BHM2	−0.14 (0.007)	0.216 (0.005)	0.257 (0.005)	1.99 (0.309)	0.807 (0.012)
	adjBHM2	−0.106 (0.012)	0.366 (0.008)	0.381 (0.009)	−44.894 (2.055)	0.765 (0.013)
	intBHM2	−0.033 (0.006)	0.177 (0.004)	0.18 (0.004)	31.376 (1.572)	1 (0)
$\theta_{3}=1$	sANOVA	−0.005 (0.008)	0.261 (0.006)	0.261 (0.006)	0.651 (1.734)	0.764 (0.013)
	sANCOVAI	−0.016 (0.013)	0.416 (0.009)	0.416 (0.009)	−58.541 (2.995)	0.742 (0.014)
	sANCOVAII	0.003 (0.006)	0.182 (0.004)	0.182 (0.004)	30.758 (1.745)	1 (0)
	reANOVA	−0.076 (0.006)	0.177 (0.004)	0.192 (0.004)	26.872 (1.184)	0.119 (0.01)
	reANCOVAI	−0.067 (0.01)	0.324 (0.007)	0.331 (0.009)	−25.881 (2.346)	0.189 (0.012)
	reANCOVAII	−0.01 (0.005)	0.144 (0.003)	0.145 (0.003)	44.903 (1.397)	0.074 (0.008)
	BHM	−0.148 (0.006)	0.186 (0.004)	0.238 (0.005)	—	0.924 (0.008)
	adjBHM	−0.104 (0.01)	0.316 (0.007)	0.333 (0.007)	−39.682 (1.902)	0.855 (0.011)
	intBHM	−0.034 (0.005)	0.158 (0.004)	0.161 (0.004)	32.317 (1.488)	1 (0)
	BHM2	−0.125 (0.006)	0.194 (0.004)	0.23 (0.005)	3.181 (0.329)	0.925 (0.008)
	adjBHM2	−0.086 (0.01)	0.325 (0.007)	0.336 (0.008)	−40.997 (2.116)	0.857 (0.011)
	intBHM2	−0.027 (0.005)	0.159 (0.004)	0.161 (0.004)	32.278 (1.517)	1 (0)
$\theta_{4}=1$	sANOVA	0.001 (0.007)	0.221 (0.005)	0.221 (0.005)	7.035 (1.72)	0.908 (0.009)
	sANCOVAI	−0.001 (0.011)	0.356 (0.008)	0.356 (0.008)	−49.518 (3.024)	0.85 (0.011)
	sANCOVAII	0.002 (0.005)	0.161 (0.004)	0.161 (0.004)	32.277 (1.675)	1 (0)
	reANOVA	−0.064 (0.006)	0.174 (0.004)	0.185 (0.004)	22.22 (1.153)	0.134 (0.011)
	reANCOVAI	−0.051 (0.009)	0.3 (0.007)	0.304 (0.007)	−27.632 (2.196)	0.195 (0.013)
	reANCOVAII	−0.007 (0.004)	0.134 (0.003)	0.135 (0.003)	43.443 (1.376)	0.073 (0.008)

When the total sample size varies from 56 to 560, the performance of these estimators is compared in Figure 1b. We can draw the following conclusions. First, only random‐effect AN(C)OVAs have issues of nonconvergence. As the total sample size increases, random‐effect AN(C)OVAs exhibit decreasing probability of nonconvergence. Second, similar to the conclusions for large sample sizes, except for random‐effect AN(C)OVAs, compared with unadjusted BHM, intBHM, and sANCOVAII reduce the RMSE on average, while adjBHM and sANCOVAI do not. As the total sample size increases, intBHM and sANCOVAII have an increasing RMSE reduction while adjBHM and sANCOVAI have a decreasing RMSE increase. In general, BHMs perform similar or better than stratified AN(C)OVAs in terms of RMSE reduction, especially for small total sample sizes. Third, except for adjBHM and sANCOVAI, all other methods control the family‐wise type I error rates at 9.75% accounting for the inflation due to multiple evaluations, computed as $1-{\left(1-0.05\right)}^{2}$, given that the individual null hypothesis type I error rate is controlled at 5%. Moreover, the family‐wise error rate becomes stable as the sample size increases. Finally, in terms of disjunctive power, it increases with the sample size for all the models except for random‐effect AN(C)OVAs, and ANCOVA II (intBHM and sANCOVAII) has the largest power, which is particularly noticeable for smaller total sample sizes.

#### 3.2.2 Results of Scenario (1) With $n_{1k}/n_{k}=0.5$

Given the results of Yang and Tsiatis [37], it is of interest to understand the efficiency of the Bayesian estimators under equal allocation, which is widely used in clinical trials.

**FIGURE 2:**
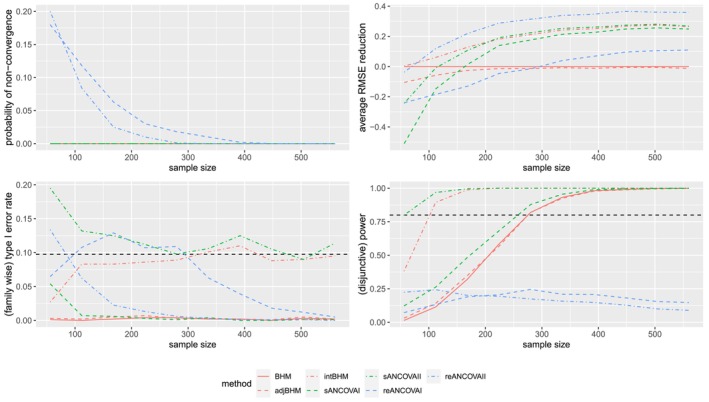
Performance of different estimators under scenario (1) with $n_{1k}/n_{k}=0.5$, where the total sample size varies. The dashed horizontal lines represent the 9.75% level of (family‐wise) type I error rate and 80% (disjunctive) power, respectively. Only seven methods (BHM, adjBHM, intBHM, sANCOVAI, sANCOVAII, reANCOVAI, reANCOVAII) are included in this figure.

**FIGURE A1:**
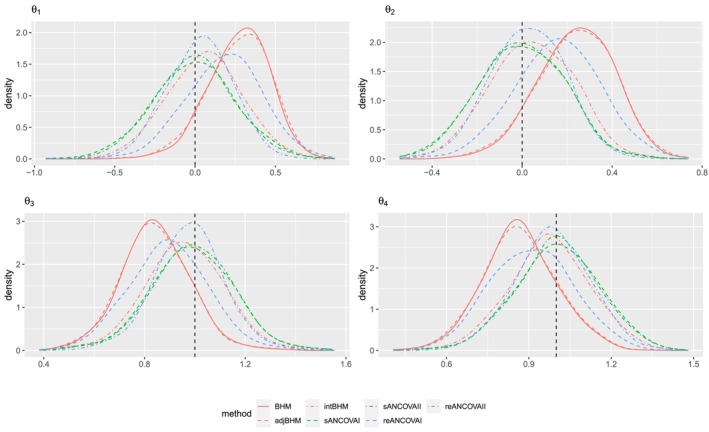
Empirical distribution of the estimator for $\theta_{k}$ under scenario (1), with $n_{1k}/n_{k}=0.5$ and n=560. Only seven methods (BHM, adjBHM, intBHM, sANCOVAI, sANCOVAII, reANCOVAI, reANCOVAII) are included in this figure.

Results under scenario (1) with $n_{1k}/n_{k}=0.5$ are shown in Figure 2, Table A1 and Figure A1 in Appendix A. Similarly to the results for $n_{1k}/n_{k}=0.75$, we observe that ANCOVA II has the smaller bias and smaller RMSE compared with unadjusted methods and ANCOVA I for BHMs and reAN(C)OVAs. For example, for large sample sizes, intBHM can reduce the RMSE values by 21%–35% compared with unadjusted BHM. Besides, intBHM also has the largest marginal power with a marginal type I error rate controlled at 5%. In addition, focusing on Bayesian estimators, adjBHM gives very similar posteriors, as well as the quantified RMSE, type I error rate, and power, to unadjusted BHM. This is because both adjBHM and the unadjusted BHM are mis‐specified models under scenario (1). In particular, even if unadjusted BHM and adjBHM have a smaller SD compared with intBHM, intBHM still has the smallest RMSE. The decrease of RMSE for intBHM is largely driven by the decrease in bias, which is benefiting from the correct specification of the true model.

#### 3.2.3 Results of Scenario (2) With $n_{1k}/n_{k}=0.5$

**FIGURE 3:**
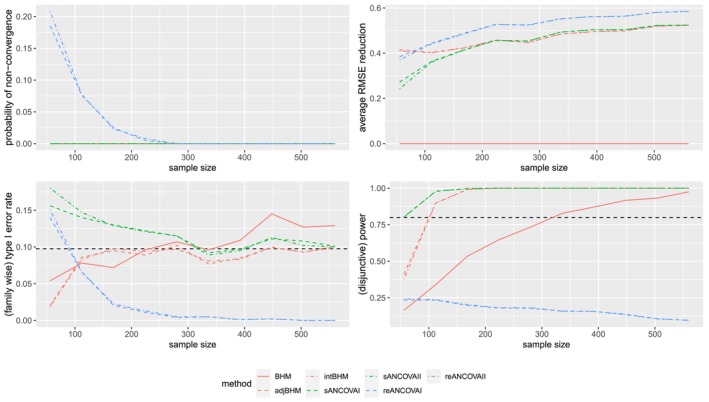
Performance of different estimators under scenario (2) with $n_{1k}/n_{k}=0.5$, where the total sample size varies. The dashed horizontal lines represent the 9.75% level of (family‐wise) type I error rate and 80% (disjunctive) power, respectively. Only seven methods (BHM, adjBHM, intBHM, sANCOVAI, sANCOVAII, reANCOVAI, reANCOVAII) are included in this figure.

**FIGURE A2:**
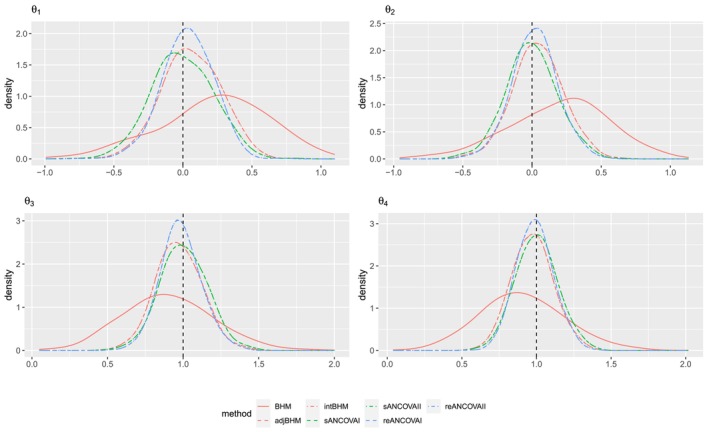
Empirical distribution of the estimator for $\theta_{k}$ under scenario (2), with $n_{1k}/n_{k}=0.5$ and n=560. Only seven methods (BHM, adjBHM, intBHM, sANCOVAI, sANCOVAII, reANCOVAI, reANCOVAII) are included in this figure.

The true model in scenario (2) is correctly specified for ANCOVA I (adjBHM, sANCOVAI, reANCOVAI) and ANCOVA II (intBHM, sANCOVAII, reANCOVAII), even if ANCOVA II requires estimating one more parameter $\gamma_{k}$. Results of scenario (2) with $n_{1k}/n_{k}=0.5$ are shown in Figure 3, Table A2 and Figure A2 in Appendix A. From these figures and tables, we can see that all the ANCOVA I methods and ANCOVA II methods perform very similarly. Compared with unadjusted methods, they have smaller bias and smaller SD. The RMSE is reduced by around 50%. Besides, covariate‐adjusted BHMs have smaller RMSE and family‐wise type I error rate, although also having lower power, than stratified ANCOVAs for small total sample sizes.

#### 3.2.4 Results of Scenario (3) With $n_{1k}/n_{k}=0.5$

**FIGURE 4:**
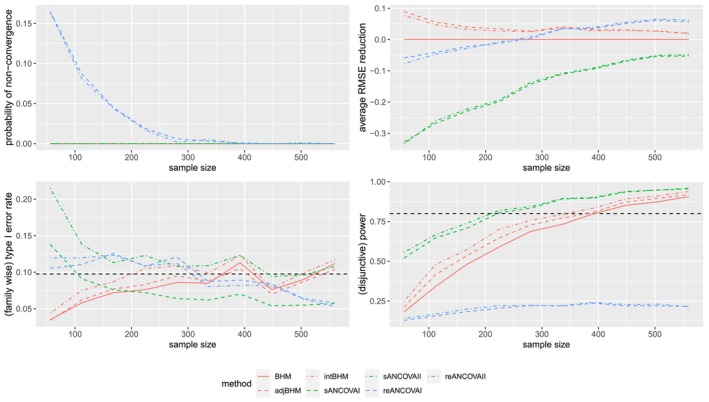
Performance of different estimators under scenario (3) with $n_{1k}/n_{k}=0.5$, where the total sample size varies. The dashed horizontal lines represent the 9.75% level of (family‐wise) type I error rate and 80% (disjunctive) power, respectively. Only seven methods (BHM, adjBHM, intBHM, sANCOVAI, sANCOVAII, reANCOVAI, reANCOVAII) are included in this figure.

**FIGURE A3:**
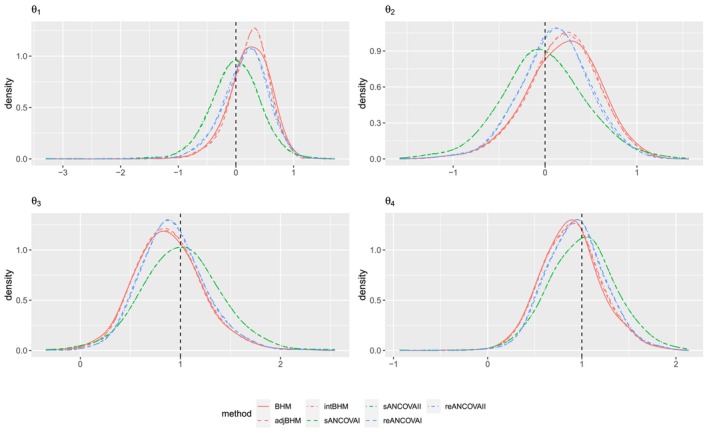
Empirical distribution of the estimator for $\theta_{k}$ under scenario (3), with $n_{1k}/n_{k}=0.5$ and n=560. Only seven methods (BHM, adjBHM, intBHM, sANCOVAI, sANCOVAII, reANCOVAI, reANCOVAII) are included in this figure.

The true model in scenario (3) is mis‐specified for all of the methods. Results of scenario (3) with $n_{1k}/n_{k}=0.5$ are shown in Figure 4, Table A3 and Figure A3 in Appendix A. First, ANCOVA I and II perform very similar to unadjusted methods in terms of bias, SD, and RMSE. Second, from Table A3 we can see that when $\theta_{1}=0$, all methods control the marginal type I error rate below 5%. However, when $\theta_{2}=0$, there is type I error rate inflation for Bayesian methods. This may be due to a larger sample size in the second subtrial ($n_{2}=120$). From Figure 4 we can see that the (family‐wise) type I error rate increases with the total trial sample size. The larger sample size can lead to stronger evidence that might not be consistent with what the half‐t prior implies. In other words, under a larger sample size, potential prior‐data conflict could inflate the type I error rate. In terms of marginal power, intBHM performs the best, followed by adjBHM. We also observe a small yet stable increase of disjunctive power for intBHM in Figure 4.

### 3.3 Summary

Overall, we can draw the following conclusions based on all the simulation results. The results of comparisons between unadjusted model and ANCOVA I or ANCOVA II models are broadly consistent across classes of methods. Comparing different classes of methods, only stratified AN(C)OVAs exhibit negligible bias, consistent with their theoretical unbiasedness, while random‐effect AN(C)OVAs show nonconvergence, resulting in a loss of power. In addition, compared with stratified AN(C)OVAs, BHMs achieve greater RMSE reduction but lower disjunctive power, particularly when the total sample size is small. This may be because, with small sample sizes, BHMs can be unstable in estimating heterogeneity and between‐subtrial variance, which can introduce bias and reduce the ability to detect signals.

Particularly, focusing on Bayesian methods, first, intBHM always has the smallest bias and largest power, and can always reduce (never increase) the RMSE compared with unadjusted BHM. Second, adjBHM can have good finite sample performance when correctly specified and for small sample sizes. However, in some scenarios, it may perform worse than unadjusted BHM, in terms of RMSE. In fact, motivated by the theoretical result of ANCOVA I in Yang and Tsiatis [37] under a frequentist framework, we do not expect adjBHM to always perform better than unadjusted BHM. In summary, we recommend intBHM, covariate adjustment with an interaction term, as it is robust to model mis‐specification and sample size.

## 4 Case Study

The MAJIC study (ISRCTN: 61925716) is a phase II randomized controlled basket trial designed to assess the long‐term comparative safety and efficacy of Ruxolitinib versus Best Available Therapy (BAT) in patients with Polycythaemia Vera (PV) or Essential Thrombocythaemia (ET), who meet the criteria for resistance or intolerance to Hydroxycarbamide therapy. For simplicity, we refer to the two subtrials as PV and ET. The JAK1/2 inhibitor Ruxolitinib, which targets Janus‐associated kinases (JAKs), specifically JAK1 and JAK2, may alleviate symptoms in patients with Polycythaemia Vera (PV) driven by JAK2 mutations, as well as in patients with Essential Thrombocythaemia (ET), whose mutations result in increased JAK2 signaling. In the PV subtrial, $n_{1}=180$ patients were randomly assigned to the Ruxolitinib treatment group ($n_{11}=93$) and BAT control group ($n_{01}=87$). Similarly, in the ET subtrial, $n_{2}=110$ patients were randomly assigned to the Ruxolitinib treatment group ($n_{12}=58$) and BAT control group ($n_{02}=52$). The primary outcome was the achievement of complete response (CR), as defined by European LeukemiaNet (ELN) criteria, within one year.

We generated hypothetical data that mimics the characteristics and the structure of the original trial, as reported in the trial results publications [48, 49]. We first randomly assign treatments and sample baseline covariates from either a normal distribution or a Bernoulli distribution, with parameters informed by Table 1 (Baseline Features at Study Entry) in Harrison et al. [49] and Table 1 (Baseline characteristics by treatment) in Harrison et al. [48]. Both Harrison et al. [49] and Harrison et al. [48] fitted multivariable logistic regression models to evaluate the effects of treatment and baseline covariates on the primary outcome. Using these results, linear models ((C1), (C2)), as detailed in Appendix C, were used to generate the continuous log‐odds ratios for PV and ET, with a signal‐to‐noise ratio of 10. The coefficients of the linear models are derived from the multivariable logistic regression results reported in Harrison et al. [49] and Harrison et al. [48].

**FIGURE 5:**
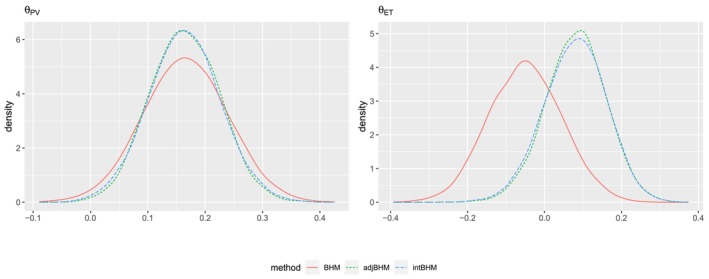
Empirical distribution of posterior samples of $\theta_{\text{PV}}$ and $\theta_{\text{ET}}$ in the analysis of a hypothetical data generated based on the MAJIC study.

We denote the log‐odds ratio as the continuous outcome of interest by $Y_{ik}$. The covariate‐adjusted estimators were obtained by including three covariates: namely, sex, JAK2V617F mutation status, and baseline haemoglobin. The parameters of interest, $\theta_{\text{PV}}$ and $\theta_{\text{ET}}$, represent the average treatment effects of Ruxolitinib versus BAT on the log‐odds ratio in the PV and ET subtrials, respectively. The data models and parameter models in BHM are multivariable extensions of the models described in Sections 2.1 and 2.2, see Equation (C3) and (C4) in the Appendix. We perform MCMC sampling using a burn‐in of 2000 with a chain of length 10 000 to sample the posteriors. The posterior means are used as point estimates, the posterior standard deviations as standard errors, and 95% credible intervals are also obtained. The results are presented in Figure 5 and Table 5.

**TABLE 5:** Estimation and inference using different methods on hypothetical data based on the MAJIC study.

		Estimate	Standard error (SE)	Credible interval	SE reduction (%)
	BHM	0.355	0.935	[−1.439,2.234]	—
$\theta_{\text{PV}}$	adjBHM	0.876	0.300	[0.303, 1.468]	67.938
	intBHM	0.884	0.313	[0.284, 1.506]	66.485
	BHM	−1.626	1.184	[−4.052,0.529]	—
$\theta_{\text{ET}}$	adjBHM	0.811	0.363	[0.067, 1.514]	69.322
	intBHM	0.805	0.373	[0.060, 1.521]	68.508

The credible intervals for both $\theta_{\text{PV}}$ and $\theta_{\text{ET}}$ using unadjusted BHM contain zero. In contrast, the credible intervals obtained from covariate‐adjusted methods indicate positive effects of ruxolitinib versus BAT in both PV and ET subtrials, demonstrating the superiority of ruxolitinib in achieving CR. Furthermore, compared to the unadjusted BHM, the covariate‐adjusted methods reduce the standard error by 66.5%–69.3%. The two covariate‐adjusted methods perform very similarly because all covariates included are prognostic factors rather than predictive factors. Additionally, we applied the same multivariable logistic regression methods (including the same three covariates as in the Bayesian analyses) used by Harrison et al. [48] and Harrison et al. [49] to analyze this hypothetical data. The estimates of $\theta_{\text{PV}}$ and $\theta_{\text{ET}}$ are 0.739 (95% CI: [0.201,1.278]) and 2.433 (95% CI: [0.512,4.353]), respectively. These results support positive effects of ruxolitinib versus BAT in both the PV and ET subtrials, consistent with the covariate‐adjusted BHMs. Specifically, the PV result is similar to that from the covariate‐adjusted BHMs, whereas the ET result shows a noticeably larger point estimate and a wider confidence interval. This is expected because this analysis is stand‐alone and the ET subtrial has a relatively small sample size.

To assess the robustness of the proposed methods, we also use CR as the outcome of interest, sampled from a Bernoulli distribution with log‐odds ratio generated by Equations (C1) and (C2). The parameter models (Equation C4) in the BHM are multivariable extensions of the models outlined in Section 2.2. We consider two types of data models: the normal multivariable linear regression models (Equation C3), and the Bernoulli multivariable logistic regression models (Equation D1), see the Appendix. The covariate‐adjusted methods using normal multivariable linear regression models yield similar results. However, those using Bernoulli multivariable logistic regression models exhibit nonconvergence of the model fitting algorithm of the logistic regression due to 0‐1 separation [50]. Detailed results are provided in Appendices C and D.

## 5 Discussion

Covariate adjustment in clinical trials is widely accepted and guided by industry standards [35, 36]. However, its application is yet to extend to basket trials where borrowing of information is a critical concern. To fill this gap, this paper explored incorporating covariate adjustment into Bayesian models to analyze basket trials for enhanced estimation precision, providing an analogue to the use of covariate adjustment in traditional randomized controlled clinical trials using frequentist methods. In particular, we propose using ANCOVA models as the data model, coupled with a conventional stipulation of parameter model, to form BHMs for borrowing of information and to estimate the treatment effects. Our simulation results demonstrate that intBHM (with treatment‐by‐covariate interaction terms) achieves the best performance in terms of bias, RMSE, and power. It is also shown to be the most robust to model misspecification. Our exploration covers settings of small to large sample sizes. Furthermore, as illustrated in Appendix B, our method is applicable to estimate the overall treatment effect in basket trials with homogenous effects. Moreover, extensive comparison of different prior beliefs is not the scope of this paper but rather to assess impact of covariate adjustment. Use of more informative priors, if not effectively discounted in situations of a prior‐data conflict, may lead to more bias for methods that permit borrowing of information. This can be perceived from comparing results of the evaluated Bayesian models with different priors (including unadjusted BHMs, adjBHMs, and intBHMs) in Table 4. In practice, when analyzing basket trials using covariate‐adjusted methods, in addition to widely recognized considerations such as variable selection and model selection, we must also account for the convergence of the MCMC algorithm. In addition, because most basket trials are currently conducted as nonrandomized (single‐arm) designs, which is recommended in the FDA guidance [8], one may also consider the applicability of our proposed methods to single‐arm basket trials. Specifically, covariate adjustment without an interaction term (ANCOVA I) could be applied to analyze single‐arm basket trials, helping to control measured confounding; however, unmeasured confounding cannot be eliminated without randomization, regardless of the analytic method. Therefore, when using covariate‐adjusted BHM to analyze single‐arm basket trials, one should be cautious about potential bias arising from unmeasured confounding.

Borrowing of information over coefficients for covariates can be a direct extension of the present work. We note advantages and drawbacks of exploiting borrowing of information on both treatment effects and other coefficients need to be adequately explored. Furthermore, extending our methods for covariate adjustment to establish more advanced Bayesian hierarchical parameter models would also be straightforward. Additional desirable properties may be attained by stipulating a parameter model like one of the EXNEX that accommodates non‐exchangeability distributions, [21], the calBHM allowing the population variance to be calibrated [28], or the discrepancy‐based approach to borrowing more strength between subtrials with high similarity than from others [30].

Another avenue of future research would be to incorporate the efficiency brought about by covariate adjustment into the trial planning stage. Explicitly, building upon the sample size determination by Zheng et al. [51], we could develop new sample size formulae for basket trials to take account of prognostic or predictive baseline covariates. Finally, we note that this paper has focused on the methodological setup and numerical simulation studies. Beyond the scope of this work, theoretical study on efficiency gains achieved by covariate‐adjusted BHMs would be among the valuable topics for further investigation.

## Author Contributions

**Jiyang Ren:** methodology, investigation, writing – original draft, writing – review and editing. **David S. Robertson:** conceptualization, investigation, methodology, writing – original draft, writing – review and editing. **Haiyan Zheng:** conceptualization, investigation, methodology, writing – original draft, writing – review and editing.

## Funding

This work was supported by the Tsinghua Scholarship for Overseas Graduate Studies (Grant No. 2023026), the UK Medical Research Council (Grant Nos. MC_UU_00002/14 and MC_UU_00040/03), and Cancer Research UK (Grant No. RCCCDF‐May24/100001).

## Conflicts of Interest

The authors declare no conflicts of interest.

## Figures and Tables

**FIGURE B1:**
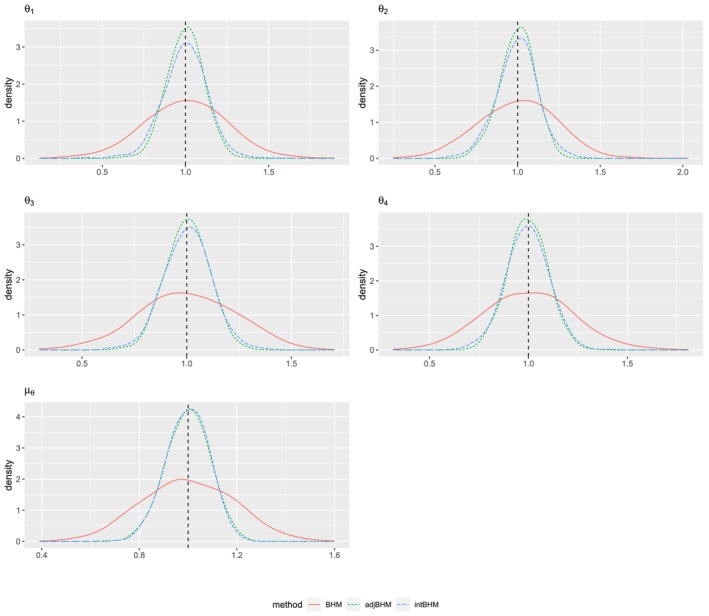
Empirical distribution of posterior means for $\theta_{k}$ and $\mu_{\theta }$, with $n_{1k}/n_{k}=0.5$ and n=560.

**FIGURE B2:**
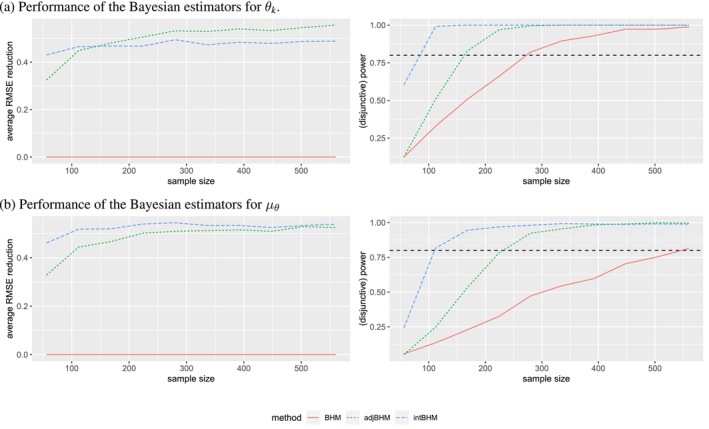
Performance of the Bayesian estimators for $\theta_{k}$ and $\mu_{\theta }$ where the total sample size varies, under scenario (4) with $n_{1k}/n_{k}=0.5$. The dashed horizontal lines represent the 80% (disjunctive) power.

**FIGURE D1:**
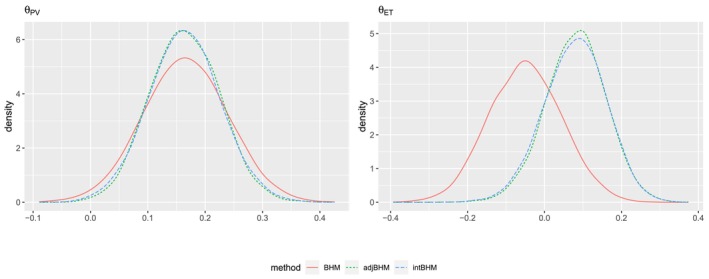
Density of posterior samples using CR as the outcome of interest and normal multivariable linear regression models as the data models.

**FIGURE D2:**
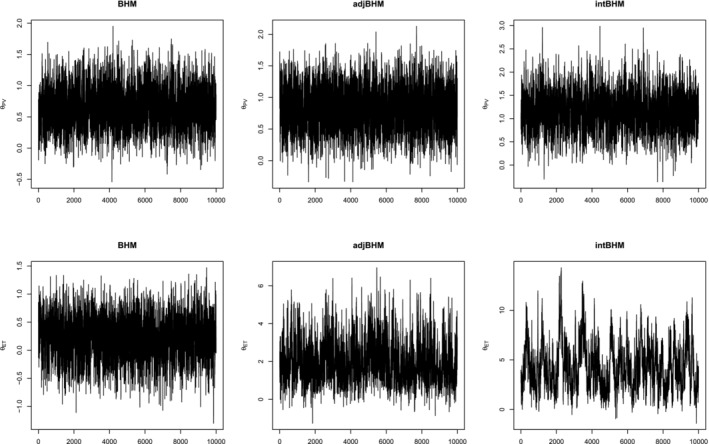
Trace plot of MCMC using CR as the outcome of interest and Bernoulli multivariable logistic regression models as the data models.

## Data Availability

The data that support the findings of this study are openly available in covariate‐adjustment‐in‐basket‐trials at: https://github.com/jiyangren/covariate‐adjustment‐in‐basket‐trials.

## Appendix A Additional Tables and Figures for the Simulation Scenario (1–3)

In this section, the simulation performance of the different estimators under scenario (1–3) with $n_{1k}/n_{k}=0.5$ and n=560 are shown in Figures A1, A2, A3 and Tables A1, A2, A3.

**TABLE A1:** Simulation performance of the different estimators of $\theta_{k}$ yielded by each BHM and frequentist method with Monte Carlo simulation error in brackets, for k=1,…,4, under scenario (1) with $n_{1k}/n_{k}=0.5$ and n=560.

		Bias	SD	RMSE	RMSE reduction (%)	Rejection Rate
	BHM	0.271 (0.006)	0.185 (0.004)	0.328 (0.005)	—	0.001 (0.001)
	adjBHM	0.272 (0.006)	0.194 (0.004)	0.334 (0.005)	−1.685 (0.531)	0.001 (0.001)
	intBHM	0.058 (0.007)	0.23 (0.005)	0.237 (0.005)	27.762 (1.269)	0.059 (0.007)
	BHM2	0.229 (0.006)	0.198 (0.004)	0.303 (0.005)	7.779 (0.291)	0 (0)
	adjBHM2	0.23 (0.007)	0.209 (0.005)	0.311 (0.005)	5.272 (0.616)	0.002 (0.001)
	intBHM2	0.047 (0.007)	0.233 (0.005)	0.237 (0.005)	27.631 (1.341)	0.056 (0.007)
$\theta_{1}=0$	sANOVA	−0.006 (0.008)	0.238 (0.005)	0.238 (0.006)	27.577 (1.674)	0.001 (0.001)
	sANCOVAI	−0.005 (0.008)	0.251 (0.006)	0.251 (0.006)	23.459 (1.808)	0.001 (0.001)
	sANCOVAII	−0.006 (0.008)	0.239 (0.005)	0.239 (0.006)	27.122 (1.673)	0.075 (0.008)
	reANOVA	0.179 (0.007)	0.226 (0.005)	0.288 (0.006)	12.201 (1.382)	0.003 (0.002)
	reANCOVAI	0.179 (0.007)	0.233 (0.005)	0.294 (0.006)	10.497 (1.475)	0.004 (0.002)
	reANCOVAII	0.029 (0.006)	0.198 (0.004)	0.2 (0.005)	38.964 (1.264)	0 (0)
	BHM	0.239 (0.005)	0.165 (0.004)	0.29 (0.004)	—	0.001 (0.001)
	adjBHM	0.239 (0.005)	0.168 (0.004)	0.293 (0.004)	−0.809 (0.473)	0.001 (0.001)
	intBHM	0.047 (0.006)	0.181 (0.004)	0.187 (0.004)	35.644 (1.055)	0.042 (0.006)
	BHM2	0.202 (0.005)	0.172 (0.004)	0.265 (0.004)	8.624 (0.269)	0.001 (0.001)
	adjBHM2	0.202 (0.006)	0.177 (0.004)	0.269 (0.005)	7.447 (0.518)	0.001 (0.001)
	intBHM2	0.039 (0.006)	0.182 (0.004)	0.186 (0.004)	35.903 (1.109)	0.042 (0.006)
$\theta_{2}=0$	sANOVA	0 (0.006)	0.182 (0.004)	0.182 (0.004)	37.212 (1.363)	0 (0)
	sANCOVAI	0 (0.006)	0.187 (0.004)	0.186 (0.004)	35.749 (1.399)	0 (0)
	sANCOVAII	−0.001 (0.006)	0.183 (0.004)	0.183 (0.004)	36.878 (1.367)	0.042 (0.006)
	reANOVA	0.157 (0.006)	0.183 (0.004)	0.242 (0.005)	16.767 (1.154)	0.003 (0.002)
	reANCOVAI	0.157 (0.006)	0.187 (0.004)	0.244 (0.005)	15.763 (1.239)	0.003 (0.002)
	reANCOVAII	0.024 (0.005)	0.162 (0.004)	0.164 (0.004)	43.579 (1.119)	0 (0)
	BHM	−0.155 (0.004)	0.134 (0.003)	0.205 (0.004)	—	0.973 (0.005)
	adjBHM	−0.158 (0.004)	0.136 (0.003)	0.208 (0.004)	−1.475 (0.462)	0.971 (0.005)
	intBHM	−0.042 (0.005)	0.156 (0.004)	0.162 (0.004)	21.148 (1.238)	1 (0)
	BHM2	−0.132 (0.004)	0.14 (0.003)	0.192 (0.004)	6.234 (0.292)	0.975 (0.005)
	adjBHM2	−0.136 (0.004)	0.142 (0.003)	0.197 (0.004)	4.192 (0.518)	0.969 (0.005)
	intBHM2	−0.036 (0.005)	0.158 (0.004)	0.162 (0.004)	21.234 (1.305)	1 (0)
$\theta_{3}=1$	sANOVA	−0.009 (0.005)	0.16 (0.004)	0.16 (0.004)	21.946 (1.567)	0.961 (0.006)
	sANCOVAI	−0.013 (0.005)	0.163 (0.004)	0.164 (0.004)	20.284 (1.588)	0.958 (0.006)
	sANCOVAII	−0.009 (0.005)	0.16 (0.004)	0.161 (0.004)	21.786 (1.567)	1 (0)
	reANOVA	−0.103 (0.005)	0.153 (0.003)	0.184 (0.004)	10.205 (1.431)	0.092 (0.009)
	reANCOVAI	−0.105 (0.005)	0.154 (0.003)	0.186 (0.004)	9.496 (1.485)	0.092 (0.009)
	reANCOVAII	‐0.024 (0.004)	0.137 (0.003)	0.139 (0.004)	32.363 (1.313)	0.059 (0.007)
	BHM	−0.133 (0.004)	0.13 (0.003)	0.186 (0.004)	—	0.995 (0.002)
	adjBHM	−0.132 (0.004)	0.133 (0.003)	0.188 (0.004)	−0.793 (0.426)	0.996 (0.002)
	intBHM	−0.025 (0.005)	0.144 (0.003)	0.146 (0.003)	21.437 (1.215)	1 (0)
	BHM2	−0.112 (0.004)	0.135 (0.003)	0.175 (0.004)	5.876 (0.28)	0.995 (0.002)
	adjBHM2	−0.112 (0.004)	0.137 (0.003)	0.177 (0.004)	5.08 (0.488)	0.995 (0.002)
	intBHM2	−0.02 (0.005)	0.145 (0.003)	0.146 (0.003)	21.534 (1.27)	1 (0)
$\theta_{4}=1$	sANOVA	0.003 (0.005)	0.146 (0.003)	0.146 (0.003)	21.669 (1.549)	0.992 (0.003)
	sANCOVAI	0.004 (0.005)	0.149 (0.003)	0.149 (0.003)	19.883 (1.577)	0.99 (0.003)
	sANCOVAII	0.003 (0.005)	0.146 (0.003)	0.146 (0.003)	21.572 (1.55)	1 (0)
	reANOVA	−0.09 (0.005)	0.143 (0.003)	0.169 (0.003)	9.345 (1.276)	0.092 (0.009)
	reANCOVAI	−0.09 (0.005)	0.146 (0.003)	0.171 (0.003)	8.134 (1.309)	0.096 (0.009)
	reANCOVAII	−0.015 (0.004)	0.132 (0.003)	0.132 (0.003)	28.847 (1.23)	0.068 (0.008)

**TABLE A2:** Simulation performance of the different estimators of $\theta_{k}$ yielded by each BHM and frequentist method with Monte Carlo simulation error in brackets, for k=1,…,4, under scenario (2) with $n_{1k}/n_{k}=0.5$ and n=560.

		Bias	SD	RMSE	RMSE reduction (%)	Rejection rate
	BHM	0.225 (0.012)	0.39 (0.009)	0.45 (0.008)	—	0.065 (0.008)
	adjBHM	0.061 (0.007)	0.212 (0.005)	0.221 (0.005)	50.968 (1.223)	0.039 (0.006)
	intBHM	0.062 (0.007)	0.212 (0.005)	0.221 (0.005)	50.939 (1.221)	0.041 (0.006)
	BHM2	0.185 (0.013)	0.414 (0.009)	0.454 (0.008)	−0.803 (0.323)	0.061 (0.008)
	adjBHM2	0.05 (0.007)	0.215 (0.005)	0.22 (0.005)	51.035 (1.237)	0.041 (0.006)
	intBHM2	0.05 (0.007)	0.215 (0.005)	0.22 (0.005)	51.047 (1.235)	0.04 (0.006)
$\theta_{1}=0$	sANOVA	−0.002 (0.016)	0.495 (0.011)	0.495 (0.01)	−9.912 (1.547)	0.057 (0.007)
	sANCOVAI	−0.003 (0.007)	0.22 (0.005)	0.22 (0.005)	51.046 (1.306)	0.044 (0.006)
	sANCOVAII	−0.003 (0.007)	0.221 (0.005)	0.221 (0.005)	51.014 (1.306)	0.044 (0.006)
	reANOVA	0.132 (0.011)	0.352 (0.008)	0.376 (0.007)	16.522 (0.707)	0.036 (0.006)
	reANCOVAI	0.035 (0.006)	0.183 (0.004)	0.186 (0.004)	58.606 (1.116)	0 (0)
	reANCOVAII	0.036 (0.006)	0.183 (0.004)	0.187 (0.004)	58.528 (1.115)	0 (0)
	BHM	0.197 (0.011)	0.357 (0.008)	0.408 (0.008)	—	0.077 (0.008)
	adjBHM	0.044 (0.006)	0.182 (0.004)	0.187 (0.004)	54.207 (1.21)	0.063 (0.008)
	intBHM	0.044 (0.006)	0.182 (0.004)	0.187 (0.004)	54.157 (1.209)	0.063 (0.008)
	BHM2	0.163 (0.012)	0.372 (0.008)	0.406 (0.008)	0.533 (0.278)	0.074 (0.008)
	adjBHM2	0.036 (0.006)	0.183 (0.004)	0.186 (0.004)	54.298 (1.218)	0.064 (0.008)
	intBHM2	0.036 (0.006)	0.183 (0.004)	0.187 (0.004)	54.241 (1.222)	0.062 (0.008)
$\theta_{2}=0$	sANOVA	0.009 (0.013)	0.409 (0.009)	0.409 (0.009)	‐0.187 (1.371)	0.054 (0.007)
	sANCOVAI	−0.003 (0.006)	0.185 (0.004)	0.185 (0.004)	54.761 (1.266)	0.059 (0.007)
	sANCOVAII	−0.003 (0.006)	0.185 (0.004)	0.185 (0.004)	54.727 (1.265)	0.058 (0.007)
	reANOVA	0.094 (0.01)	0.328 (0.007)	0.341 (0.007)	16.375 (0.757)	0.033 (0.006)
	reANCOVAI	0.018 (0.005)	0.163 (0.004)	0.164 (0.004)	59.795 (1.12)	0 (0)
	reANCOVAII	0.018 (0.005)	0.163 (0.004)	0.164 (0.004)	59.707 (1.123)	0 (0)
	BHM	−0.098 (0.009)	0.296 (0.007)	0.312 (0.007)	—	0.852 (0.011)
	adjBHM	−0.029 (0.005)	0.151 (0.003)	0.153 (0.003)	50.852 (1.401)	1 (0)
	intBHM	−0.029 (0.005)	0.151 (0.003)	0.153 (0.003)	50.831 (1.402)	1 (0)
	BHM2	−0.077 (0.01)	0.308 (0.007)	0.317 (0.007)	−1.517 (0.267)	0.847 (0.011)
	adjBHM2	−0.023 (0.005)	0.152 (0.003)	0.153 (0.004)	50.819 (1.409)	1 (0)
	intBHM2	−0.023 (0.005)	0.152 (0.003)	0.154 (0.004)	50.737 (1.413)	1 (0)
$\theta_{3}=1$	sANOVA	0.023 (0.011)	0.346 (0.008)	0.347 (0.008)	‐11.048 (1.289)	0.824 (0.012)
	sANCOVAI	0.004 (0.005)	0.154 (0.003)	0.154 (0.004)	50.62 (1.455)	1 (0)
	sANCOVAII	0.004 (0.005)	0.154 (0.003)	0.154 (0.004)	50.594 (1.456)	1 (0)
	reANOVA	−0.048 (0.009)	0.27 (0.006)	0.274 (0.006)	12.195 (0.716)	0.143 (0.011)
	reANCOVAI	−0.016 (0.004)	0.134 (0.003)	0.135 (0.003)	56.868 (1.287)	0.062 (0.008)
	reANCOVAII	−0.016 (0.004)	0.134 (0.003)	0.135 (0.003)	56.775 (1.286)	0.061 (0.008)
	BHM	−0.094 (0.009)	0.282 (0.006)	0.297 (0.006)	—	0.903 (0.009)
	adjBHM	−0.027 (0.004)	0.137 (0.003)	0.139 (0.003)	53.208 (1.286)	1 (0)
	intBHM	−0.027 (0.004)	0.137 (0.003)	0.139 (0.003)	53.218 (1.285)	1 (0)
	BHM2	−0.075 (0.009)	0.291 (0.007)	0.3 (0.007)	−0.979 (0.236)	0.899 (0.01)
	adjBHM2	−0.022 (0.004)	0.137 (0.003)	0.139 (0.003)	53.267 (1.289)	1 (0)
	intBHM2	−0.022 (0.004)	0.137 (0.003)	0.139 (0.003)	53.208 (1.291)	1 (0)
$\theta_{4}=1$	sANOVA	0.012 (0.01)	0.318 (0.007)	0.318 (0.007)	−7.002 (1.148)	0.893 (0.01)
	sANCOVAI	0 (0.004)	0.139 (0.003)	0.139 (0.003)	53.305 (1.316)	1 (0)
	sANCOVAII	0 (0.004)	0.139 (0.003)	0.139 (0.003)	53.3 (1.316)	1 (0)
	reANOVA	−0.037 (0.008)	0.26 (0.006)	0.262 (0.006)	11.77 (0.658)	0.167 (0.012)
	reANCOVAI	−0.012 (0.004)	0.123 (0.003)	0.123 (0.003)	58.607 (1.151)	0.066 (0.008)
	reANCOVAII	−0.012 (0.004)	0.123 (0.003)	0.123 (0.003)	58.607 (1.149)	0.065 (0.008)

**TABLE A3:** Simulation performance of the different estimators of $\theta_{k}$ yielded by each BHM and frequentist method with Monte Carlo simulation error in brackets, for k=1,…,4, under scenario (3) with $n_{1k}/n_{k}=0.5$ and n=560.

		Bias	SD	RMSE	RMSE reduction (%)	Rejection Rate
	BHM	0.242 (0.011)	0.353 (0.008)	0.428 (0.008)	—	0.039 (0.006)
	adjBHM	0.238 (0.011)	0.352 (0.008)	0.425 (0.012)	0.579 (1.869)	0.032 (0.006)
	intBHM	0.226 (0.011)	0.355 (0.008)	0.421 (0.012)	1.533 (1.699)	0.038 (0.006)
	BHM2	0.197 (0.012)	0.378 (0.008)	0.426 (0.009)	0.418 (0.468)	0.029 (0.005)
	adjBHM2	0.195 (0.012)	0.375 (0.008)	0.422 (0.014)	1.262 (2.293)	0.029 (0.005)
	intBHM2	0.185 (0.012)	0.377 (0.008)	0.42 (0.014)	1.866 (2.17)	0.032 (0.006)
$\theta_{1}=0$	sANOVA	−0.018 (0.015)	0.459 (0.01)	0.46 (0.012)	−7.461 (2.137)	0.019 (0.004)
	sANCOVAI	−0.012 (0.014)	0.444 (0.01)	0.444 (0.017)	−3.761 (3.404)	0.014 (0.004)
	sANCOVAII	−0.011 (0.014)	0.438 (0.01)	0.438 (0.016)	−2.436 (3.094)	0.053 (0.007)
	reANOVA	0.168 (0.013)	0.398 (0.009)	0.432 (0.01)	−1.004 (1.25)	0.053 (0.007)
	reANCOVAI	0.164 (0.013)	0.406 (0.009)	0.437 (0.013)	−2.226 (2.324)	0.051 (0.007)
	reANCOVAII	0.151 (0.013)	0.403 (0.009)	0.431 (0.014)	−0.719 (2.356)	0.044 (0.006)
	BHM	0.193 (0.012)	0.392 (0.009)	0.437 (0.009)	—	0.082 (0.009)
	adjBHM	0.19 (0.012)	0.379 (0.008)	0.424 (0.009)	2.948 (0.684)	0.079 (0.009)
	intBHM	0.177 (0.012)	0.384 (0.009)	0.422 (0.009)	3.348 (0.695)	0.09 (0.009)
	BHM2	0.154 (0.013)	0.411 (0.009)	0.439 (0.009)	−0.434 (0.336)	0.07 (0.008)
	adjBHM2	0.151 (0.013)	0.397 (0.009)	0.424 (0.009)	2.848 (0.782)	0.071 (0.008)
	intBHM2	0.141 (0.013)	0.4 (0.009)	0.424 (0.009)	3.01 (0.805)	0.074 (0.008)
$\theta_{2}=0$	sANOVA	−0.02 (0.015)	0.469 (0.01)	0.469 (0.011)	−7.399 (1.521)	0.045 (0.007)
	sANCOVAI	−0.017 (0.014)	0.45 (0.01)	0.45 (0.011)	−2.922 (1.652)	0.044 (0.006)
	sANCOVAII	−0.017 (0.014)	0.449 (0.01)	0.449 (0.011)	−2.807 (1.647)	0.057 (0.007)
	reANOVA	0.114 (0.012)	0.389 (0.009)	0.405 (0.009)	7.187 (0.867)	0.051 (0.007)
	reANCOVAI	0.112 (0.012)	0.373 (0.008)	0.389 (0.009)	10.985 (1.049)	0.049 (0.007)
	reANCOVAII	0.1 (0.012)	0.375 (0.008)	0.388 (0.009)	11.279 (1.108)	0.043 (0.006)
	BHM	−0.12 (0.011)	0.336 (0.008)	0.357 (0.008)	—	0.742 (0.014)
	adjBHM	−0.117 (0.01)	0.328 (0.007)	0.349 (0.008)	2.22 (0.732)	0.78 (0.013)
	intBHM	−0.11 (0.011)	0.332 (0.007)	0.35 (0.008)	1.914 (0.743)	0.804 (0.013)
	BHM2	−0.095 (0.011)	0.351 (0.008)	0.363 (0.009)	−1.952 (0.304)	0.743 (0.014)
	adjBHM2	−0.094 (0.011)	0.342 (0.008)	0.355 (0.009)	0.52 (0.813)	0.778 (0.013)
	intBHM2	−0.088 (0.011)	0.346 (0.008)	0.357 (0.009)	−0.034 (0.844)	0.795 (0.013)
$\theta_{3}=1$	sANOVA	0.015 (0.013)	0.402 (0.009)	0.402 (0.01)	−12.884 (1.397)	0.755 (0.014)
	sANCOVAI	0.012 (0.012)	0.389 (0.009)	0.389 (0.01)	−9.169 (1.568)	0.776 (0.013)
	sANCOVAII	0.012 (0.012)	0.389 (0.009)	0.389 (0.01)	−9.11 (1.573)	0.781 (0.013)
	reANOVA	−0.083 (0.01)	0.331 (0.007)	0.341 (0.008)	4.376 (0.798)	0.154 (0.011)
	reANCOVAI	−0.079 (0.01)	0.321 (0.007)	0.33 (0.008)	7.329 (1.078)	0.155 (0.011)
	reANCOVAII	−0.073 (0.01)	0.322 (0.007)	0.33 (0.008)	7.504 (1.117)	0.15 (0.011)
	BHM	−0.117 (0.01)	0.313 (0.007)	0.335 (0.008)	—	0.819 (0.012)
	adjBHM	−0.112 (0.01)	0.31 (0.007)	0.329 (0.008)	1.536 (0.656)	0.838 (0.012)
	intBHM	−0.105 (0.01)	0.312 (0.007)	0.329 (0.008)	1.526 (0.682)	0.861 (0.011)
	BHM2	−0.095 (0.01)	0.324 (0.007)	0.338 (0.008)	−0.97 (0.292)	0.82 (0.012)
	adjBHM2	−0.091 (0.01)	0.32 (0.007)	0.332 (0.008)	0.656 (0.734)	0.839 (0.012)
	intBHM2	−0.085 (0.01)	0.323 (0.007)	0.334 (0.008)	0.185 (0.769)	0.858 (0.011)
$\theta_{4}=1$	sANOVA	0.005 (0.011)	0.359 (0.008)	0.359 (0.01)	−7.347 (1.342)	0.817 (0.012)
	sANCOVAI	0.005 (0.011)	0.353 (0.008)	0.352 (0.01)	−5.343 (1.503)	0.832 (0.012)
	sANCOVAII	0.005 (0.011)	0.352 (0.008)	0.352 (0.01)	−5.304 (1.509)	0.839 (0.012)
	reANOVA	−0.072 (0.01)	0.311 (0.007)	0.319 (0.008)	4.642 (0.856)	0.185 (0.012)
	reANCOVAI	−0.068 (0.01)	0.306 (0.007)	0.314 (0.008)	6.259 (1.073)	0.166 (0.012)
	reANCOVAII	−0.061 (0.01)	0.306 (0.007)	0.312 (0.008)	6.737 (1.101)	0.164 (0.012)

## Appendix B Simulation Study With Homogenous Effects, Scenario (4)

### Aims

We aim to compare the performance of covariate‐adjusted with unadjusted BHM on estimating both subtrial‐specific treatment effects and overall treatment effect, considering the impact of different sample sizes, under a homogenous scenario and adjusting for more than one covariate.

### Data‐generating Mechanisms

Consider a basket trial with K=4 subtrials. The sample size ratio of different subtrials is $n_{1}:n_{2}:n_{3}:n_{4}=2:3:4:5$ with $n_{1k}/n_{k}=0.5,\forall k=1,\ldots ,4$. A range of different sample sizes are set for the numerical evaluation so that the total sample size, $n=\sum_{k=1}^{K}n_{k}$, ranges from 56 to 560 in increments of 56. We let the treatment effect θ=(1,1,1,1), and two‐dimensional pretreatment covariates, $X_{ik1}\overset{i.i.d.}{∼}N(0,1),X_{ik2}\overset{i.i.d.}{∼}N(0,1),\forall k=1,\ldots ,4$. We generate the outcomes from the formula $Y_{ik}=1+\theta_{k}T_{ik}-2X_{ik1}-2X_{ik2}+4T_{ik}X_{ik1}+\epsilon_{ik}$, where $X_{ik1}$ is considered as predictive factor and $X_{ik2}$ is considered as prognostic factor, $\epsilon_{ik}$ are i.i.d. standard normal random noise.

### Estimands

Our estimands are subtrial‐specific treatment effects $\theta_{k}$ and overall treatment effect $\mu_{\theta }$.

### Methods

Each of the three BHMs is fitted to the simulated trial data, so as to compare the performance of different methods. As noted earlier, we use cluster‐centered covariates $X_{ik1}-{\bar{X}}_{k1}$ and $X_{ik2}-{\bar{X}}_{k2}$ to perform covariate adjustment. We set an inverse−Gamma(2,20) prior on the variance of the outcome, a N(0,100) prior on $\mu_{\theta }$, $\alpha_{k}$, $\beta_{k1}$ (coefficient of $X_{ik1}-{\bar{X}}_{k1}$), $\beta_{k2}$ (coefficient of $X_{ik2}-{\bar{X}}_{k2}$), $\gamma_{k1}$ (coefficient of the interaction term of $T_{ik}$ and $X_{ik1}-{\bar{X}}_{k1}$), $\gamma_{k2}$ (coefficient of the interaction term of $T_{ik}$ and $X_{ik2}-{\bar{X}}_{k2}$), and a HT(2,5) prior on $\sigma_{\theta }$. We apply MCMC using a burn‐in of 2000 with a chain of length 10,000 to sample the posteriors and use posterior means, with 95% credible intervals symmetric about the posterior mean, to estimate $\theta_{k}$ and common mean $\mu_{\theta }$.

### Performance Measures

We simulate B=1000 replicates of basket trials following the configuration above to generate the trial data. We compare the performance of three models in terms of the metrics and their MCSEs defined in Table 3.

### Exploration and Visualization of Results

The results are presented in Figures B1, B2, and Table B1. From Figure B1 and Table B1, we observe that for large sample sizes, the biases of all three BHMs are negligible in this homogenous scenario. Second, the estimator of $\theta_{k}$ using adjBHM demonstrates the smallest RMSE, reducing the RMSE by 55% compared with unadjusted BHM when n=560. This may be because, under a homogeneous setting, including interaction terms (which assumes the treatment effects vary across covariate‐by‐basket) allows effects to drift more freely, weakening cross‐basket borrowing and thereby increasing variance. When estimating the overall effect $\mu_{\theta }$, adjBHM and intBHM perform very similar, better than unadjusted method. Moreover, both adjBHM and intBHM have sufficient power.

From Figure B2, we could conclude similar results with other simulation scenarios. As the total sample size increases, both adjBHM and intBHM exhibit increasing RMSE reduction and disjunctive power.

**TABLE B1:** Simulation performance of the Bayesian estimators of $\theta_{k}$ and $\mu_{\theta }$ with Monte Carlo simulation error in brackets, for k=1,…,4, under scenario (4) with $n_{1k}/n_{k}=0.5$ and n=560.

		Bias	SD	RMSE	RMSE reduction (%)	Rejection Rate
	BHM	0 (0.008)	0.244 (0.005)	0.244 (0.006)	—	0.671 (0.015)
$\theta_{1}=1$	adjBHM	0 (0.003)	0.108 (0.002)	0.108 (0.003)	55.956 (1.296)	0.981 (0.004)
	intBHM	−0.002 (0.004)	0.133 (0.003)	0.133 (0.004)	45.593 (1.946)	0.997 (0.002)
	BHM	0.001 (0.007)	0.233 (0.005)	0.233 (0.005)	—	0.789 (0.013)
$\theta_{2}=1$	adjBHM	−0.002 (0.003)	0.105 (0.002)	0.105 (0.002)	55.04 (1.372)	0.998 (0.001)
	intBHM	−0.003 (0.004)	0.122 (0.003)	0.122 (0.003)	47.627 (1.699)	1 (0)
	BHM	0.003 (0.007)	0.227 (0.005)	0.227 (0.005)	—	0.874 (0.01)
$\theta_{3}=1$	adjBHM	0.003 (0.003)	0.101 (0.002)	0.101 (0.002)	55.427 (1.284)	1 (0)
	intBHM	0.004 (0.004)	0.111 (0.002)	0.111 (0.003)	50.877 (1.499)	1 (0)
	BHM	0.002 (0.007)	0.223 (0.005)	0.223 (0.005)	—	0.907 (0.009)
$\theta_{4}=1$	adjBHM	−0.003 (0.003)	0.098 (0.002)	0.098 (0.002)	56.028 (1.327)	1 (0)
	intBHM	−0.005 (0.003)	0.108 (0.002)	0.108 (0.003)	51.345 (1.543)	1 (0)
	BHM	0 (0.006)	0.185 (0.004)	0.185 (0.004)	—	0.814 (0.012)
$\mu_{\theta }=1$	adjBHM	−0.001 (0.003)	0.088 (0.002)	0.088 (0.002)	52.453 (1.347)	0.996 (0.002)
	intBHM	−0.002 (0.003)	0.086 (0.002)	0.086 (0.002)	53.84 (1.29)	0.989 (0.003)

## Appendix C More Details of Case Study

To generate the log odds ratio, the continuous outcome of interest $Y_{ik}$, we consider the following covariates: age ($X_{ik1}$), haemoglobin ($X_{ik2}$), number of previous therapy ($X_{ik3}$), sex ($X_{ik4}$), previous thrombosis ($X_{ik5}$), whether intolerant to hydroxycarbomide ($X_{ik6}$), whether intolerant and resistant to hydroxycarbomide ($X_{ik7}$), splenomegaly at baseline ($X_{ik8}$), splenectomy at baseline ($X_{ik9}$), white blood cells ($W_{ik1}$), whether platelets count in the middle range ($W_{ik2}$), whether platelets count in the upper range ($W_{ik3}$), whether JAK2 mutation status is CALR positive ($W_{ik4}$), whether JAK2 mutation status is JAK2V617F postive ($W_{ik5}$).

We use the following linear model to create the continuous log‐odds ratios for PV (k=1) and ET (k=2).

(C1)
$$\begin{array}{ll} Y_{i1} & =\log(63/117)+\log(2.03)T_{i1}+\log(1.01)X_{i11}+\log(1.02)X_{i12}\\ & \;+\log(0.77)X_{i13}+\log(0.97)X_{i14}+\log(0.63)X_{i15}\\ & \;+\log(0.94)X_{i16}+\log(0.7)X_{i17}+\log(0.13)X_{i18}\\ & \;+\log(1.26)X_{i19}, \end{array}$$

(C2)
$$\begin{array}{ll} Y_{i2} & =\log(50/60)+\log(1.14)T_{i2}+\log(1.82)X_{i22}+\log(1.17)X_{i26}\\ & \;+\log(1.45)W_{i21}+\log(0.58)W_{i22}+\log(0.44)W_{i23}\\ & \;+\log(0.9)W_{i24}+\log(0.79)(1-W_{i24}-W_{i25}), \end{array}$$

where the coefficients of these two linear models are derived from the multivariable logistic regression results reported in Harrison et al. [49] and Harrison et al. [48].

The data models are

(C3)
$$\begin{array}{ll} Y_{ik} & ∼N(\alpha_{k}+\theta_{k}T_{ik},\sigma_{y}^{2}),\\ Y_{ik} & ∼N(\alpha_{k}+\theta_{k}T_{ik}+\beta_{k2}X_{ik2}+\beta_{k4}X_{ik4}+\beta_{k5}W_{ik5},\sigma_{y}^{2}),\\ Y_{ik} & ∼N(\alpha_{k}+\theta_{k}T_{ik}+\beta_{k2}X_{ik2}+\beta_{k4}X_{ik4}+\beta_{k5}W_{ik5}\\ & \;+T_{ik}(\gamma_{k2}X_{ik2}+\gamma_{k4}X_{ik4}+\gamma_{k5}W_{ik5}),\sigma_{y}^{2}), \end{array}$$

for unadjusted BHM, adjBHM and intBHM, respectively. The parameter models are

(C4)
$$\begin{array}{ll} \alpha_{k},\beta_{k2},\beta_{k4},\beta_{k5},\gamma_{k2},\gamma_{k4},\gamma_{k5} & ∼N(0,100)\\ \sigma_{y}^{2} & ∼inverse-Gamma(2,20)\\ \theta_{k}|\mu_{\theta },\sigma_{\theta } & ∼N(\mu_{\theta },\sigma_{\theta }^{2})\\ \mu_{\theta } & ∼N(0,100)\\ \sigma_{\theta } & ∼HT(2,5). \end{array}$$

## Appendix D Sensitivity Analysis of Case Study

The outcome of interest is CR in this section, sampled from a Bernoulli distribution with log‐odds ratio generated by Equations (C1) and (C2).

We consider two types of data models:

- the normal multivariable linear regression models (Equation C3)
- the Bernoulli multivariable logistic regression models
  (D1)
  $$\begin{array}{ll} Y_{ik} & ∼\text{Bernoulli}(p_{ik}),\\ \log\{p_{ik}/(1-p_{ik})\} & =\alpha_{k}+\theta_{k}T_{ik},\\ \log\{p_{ik}/(1-p_{ik})\} & =\alpha_{k}+\theta_{k}T_{ik}+\beta_{k2}X_{ik2}+\beta_{k4}X_{ik4}\\ & \;+\beta_{k5}W_{ik5},\\ \log\{p_{ik}/(1-p_{ik})\} & =\alpha_{k}+\theta_{k}T_{ik}+\beta_{k2}X_{ik2}+\beta_{k4}X_{ik4}\\ & \;+\beta_{k5}W_{ik5}\\ & \;+T_{ik}(\gamma_{k2}X_{ik2}+\gamma_{k4}X_{ik4}+\gamma_{k5}W_{ik5}), \end{array}$$
  for unadjusted BHM, adjBHM and intBHM, respectively.

The parameter models are the same as those in Section B (Equation C4). The results of using CR as the outcome of interest are shown in Figures D1, D2 and Table D1.

It can be observed that when using CR as the outcome of interest and normal multivariable linear regression models as the data models, all of the credible intervals for $\theta_{\text{ET}}$ and the credible interval for $\theta_{\text{PV}}$ under the unadjusted BHM contain zero. However, the credible intervals for $\theta_{\text{PV}}$ obtained using covariate‐adjusted methods show positive effects of ruxolitinib versus BAT, demonstrating the superiority of CR for ruxolitinib in the PV subtrial. Besides, compared with the unadjusted BHM, covariate‐adjusted methods show the ability to reduce the standard error by 9.6%–11.8%. These findings suggest that even when the parameter models are mis‐specified, the proposed BHM methods remain robust.

However, when using CR as the outcome of interest and Bernoulli multivariable logistic regression models as the data models, the model fitting algorithm of the logistic regression does not converge for the ET subtrial. As is widely known, the estimators of logistic regression coefficients may fail to be unique or may not exist when the outcomes (1 and 0 s) can be perfectly separated by a hyperplane [50]. Therefore, one should be cautious in practice when applying logistic regression models.

**TABLE D1:** Estimation and inference using CR as the outcome of interest and normal multivariable linear regression models as the data models.

		Estimate	Standard error (SE)	Credible interval	SE reduction (%)
	BHM	0.152	0.088	[−0.019,0.332]	—
$\theta_{\text{PV}}$	adjBHM	0.159	0.080	[0.002, 0.316]	9.625
	intBHM	0.160	0.078	[0.007, 0.315]	11.762
	BHM	0.042	0.110	[−0.184,0.245]	—
$\theta_{\text{ET}}$	adjBHM	0.161	0.099	[−0.033,0.356]	10.334
	intBHM	0.161	0.099	[−0.033,0.359]	10.168

## References

[1] E. A. Ashley , “Towards Precision Medicine,” Nature Reviews Genetics 17, no. 9 (2016): 507–522. 10.1038/nrg.2016.86 27528417

[2] S. E. Jackson and J. D. Chester , “Personalised Cancer Medicine,” International Journal of Cancer 137, no. 2 (2015): 262–266. 24789362 10.1002/ijc.28940

[3] N. J. Schork , “Personalized Medicine: Time for One‐Person Trials,” Nature 520, no. 7549 (2015): 609–611. 25925459 10.1038/520609a

[4] D. M. Hyman , B. S. Taylor , and J. Baselga , “Implementing Genome‐Driven Oncology,” Cell 168, no. 4 (2017): 584–599. 28187282 10.1016/j.cell.2016.12.015 PMC5463457

[5] J. J. Tao , A. M. Schram , and D. M. Hyman , “Basket Studies: Redefining Clinical Trials in the Era of Genome‐Driven Oncology,” Annual Review of Medicine 69, no. 1 (2018): 319–331. 10.1146/annurev-med-062016-050343 PMC7455011 29120700

[6] J. Woodcock and L. M. LaVange , “Master Protocols to Study Multiple Therapies, Multiple Diseases, or Both,” New England Journal of Medicine 377, no. 1 (2017): 62–70. 28679092 10.1056/NEJMra1510062

[7] L. Renfro and D. Sargent , “Statistical Controversies in Clinical Research: Basket Trials, Umbrella Trials, and Other Master Protocols: A Review and Examples,” Annals of Oncology 28, no. 1 (2017): 34–43. 28177494 10.1093/annonc/mdw413 PMC5834138

[8] FDA , “Master Protocols: Efficient Clinical Trial Design Strategies to Expedite Development of Oncology Drugs and Biologics. Guidance for Industry,” 2022.

[9] D. M. Hyman , I. Puzanov , V. Subbiah , et al., “Vemurafenib in Multiple Nonmelanoma Cancers With BRAF V600 Mutations,” New England Journal of Medicine 373, no. 8 (2015): 726–736. 26287849 10.1056/NEJMoa1502309 PMC4971773

[10] K. M. Cunanan , A. Iasonos , R. Shen , C. B. Begg , and M. Gönen , “An Efficient Basket Trial Design,” Statistics in Medicine 36, no. 10 (2017): 1568–1579. 28098411 10.1002/sim.7227 PMC5380524

[11] B. P. Hobbs , R. C. Pestana , E. C. Zabor , A. M. Kaizer , and D. S. Hong , “Basket Trials: Review of Current Practice and Innovations for Future Trials,” Journal of Clinical Oncology 40, no. 30 (2022): 3520–3528. 35537102 10.1200/JCO.21.02285 PMC10476732

[12] K. T. Flaherty , I. Puzanov , K. B. Kim , et al., “Inhibition of Mutated, Activated BRAF in Metastatic Melanoma,” New England Journal of Medicine 363, no. 9 (2010): 809–819. 20818844 10.1056/NEJMoa1002011 PMC3724529

[13] E. Tiacci , V. Trifonov , G. Schiavoni , et al., “BRAF Mutations in Hairy‐Cell Leukemia,” New England Journal of Medicine 364, no. 24 (2011): 2305–2315. 21663470 10.1056/NEJMoa1014209 PMC3689585

[14] A. Prahallad , C. Sun , S. Huang , et al., “Unresponsiveness of Colon Cancer to BRAF (V600E) Inhibition Through Feedback Activation of EGFR,” Nature 483, no. 7387 (2012): 100–103. 22281684 10.1038/nature10868

[15] A. C. Palmer , D. Plana , and P. K. Sorger , “Comparing the Efficacy of Cancer Therapies Between Subgroups in Basket Trials,” Cell Systems 11, no. 5 (2020): 449–460. 33220857 10.1016/j.cels.2020.09.003 PMC8022348

[16] C. Chen , X. Li , S. Yuan , Z. Antonijevic , R. Kalamegham , and R. A. Beckman , “Statistical Design and Considerations of a Phase 3 Basket Trial for Simultaneous Investigation of Multiple Tumor Types in One Study,” Statistics in Biopharmaceutical Research 8, no. 3 (2016): 248–257.

[17] W. Li , J. Zhao , X. Li , C. Chen , and R. A. Beckman , “Multi‐Stage Enrichment and Basket Trial Designs With Population Selection,” Statistics in Medicine 38, no. 29 (2019): 5470–5485. 31621949 10.1002/sim.8371

[18] H. Zhou , F. Liu , C. Wu , E. H. Rubin , V. L. Giranda , and C. Chen , “Optimal Two‐Stage Designs for Exploratory Basket Trials,” Contemporary Clinical Trials 85 (2019): 105807. 31260789 10.1016/j.cct.2019.06.021

[19] P. F. Thall , J. K. Wathen , B. N. Bekele , R. E. Champlin , L. H. Baker , and R. S. Benjamin , “Hierarchical Bayesian Approaches to Phase II Trials in Diseases With Multiple Subtypes,” Statistics in Medicine 22, no. 5 (2003): 763–780. 12587104 10.1002/sim.1399

[20] S. M. Berry , K. R. Broglio , S. Groshen , and D. A. Berry , “Bayesian Hierarchical Modeling of Patient Subpopulations: Efficient Designs of Phase II Oncology Clinical Trials,” Clinical Trials 10, no. 5 (2013): 720–734. 23983156 10.1177/1740774513497539 PMC4319656

[21] B. Neuenschwander , S. Wandel , S. Roychoudhury , and S. Bailey , “Robust Exchangeability Designs for Early Phase Clinical Trials With Multiple Strata,” Pharmaceutical Statistics 15, no. 2 (2016): 123–134. 26685103 10.1002/pst.1730

[22] R. Liu , Z. Liu , M. Ghadessi , and R. Vonk , “Increasing the Efficiency of Oncology Basket Trials Using a Bayesian Approach,” Contemporary Clinical Trials 63 (2017): 67–72. 28629993 10.1016/j.cct.2017.06.009

[23] Y. Chu and Y. Yuan , “BLAST: Bayesian Latent Subgroup Design for Basket Trials Accounting for Patient Heterogeneity,” Journal of the Royal Statistical Society: Series C: Applied Statistics 67, no. 3 (2018): 723–740.

[24] J. Jin , Q. Liu , W. Zheng , et al., “A Bayesian Method for the Detection of Proof of Concept in Early Phase Oncology Studies With a Basket Design,” Statistics in Biosciences 12 (2020): 167–179.

[25] N. Chen and J. J. Lee , “Bayesian Hierarchical Classification and Information Sharing for Clinical Trials With Subgroups and Binary Outcomes,” Biometrical Journal 61, no. 5 (2019): 1219–1231. 30506747 10.1002/bimj.201700275 PMC6546564

[26] N. Chen and J. J. Lee , “Bayesian Cluster Hierarchical Model for Subgroup Borrowing in the Design and Analysis of Basket Trials With Binary Endpoints,” Statistical Methods in Medical Research 29, no. 9 (2020): 2717–2732. 32178585 10.1177/0962280220910186

[27] L. Jiang , R. Li , F. Yan , T. A. Yap , and Y. Yuan , “Shotgun: A Bayesian Seamless Phase I‐II Design to Accelerate the Development of Targeted Therapies and Immunotherapy,” Contemporary Clinical Trials 104 (2021): 106338. 33711459 10.1016/j.cct.2021.106338 PMC8180491

[28] Y. Chu and Y. Yuan , “A Bayesian Basket Trial Design Using a Calibrated Bayesian Hierarchical Model,” Clinical Trials 15, no. 2 (2018): 149–158. 29499621 10.1177/1740774518755122 PMC5891374

[29] J. Jin , M. K. Riviere , X. Luo , and Y. Dong , “Bayesian Methods for the Analysis of Early‐Phase Oncology Basket Trials With Information Borrowing Across Cancer Types,” Statistics in Medicine 39, no. 25 (2020): 3459–3475. 32717103 10.1002/sim.8675

[30] H. Zheng and J. M. Wason , “Borrowing of Information Across Patient Subgroups in a Basket Trial Based on Distributional Discrepancy,” Biostatistics 23, no. 1 (2022): 120–135. 32380518 10.1093/biostatistics/kxaa019 PMC8759447

[31] J. Asano and A. Hirakawa , “A Bayesian Basket Trial Design Accounting for Uncertainties of Homogeneity and Heterogeneity of Treatment Effect Among Subpopulations,” Pharmaceutical Statistics 19, no. 6 (2020): 975–1000. 32779393 10.1002/pst.2049

[32] R. Simon , S. Geyer , J. Subramanian , and S. Roychowdhury , “The Bayesian Basket Design for Genomic Variant‐Driven Phase II Trials,” Seminars in Oncology 43, no. 1 (2016): 13–18. 26970120 10.1053/j.seminoncol.2016.01.002

[33] M. A. Psioda , J. Xu , Q. Jiang , C. Ke , Z. Yang , and J. G. Ibrahim , “Bayesian adaptive basket trial design using model averaging,” Biostatistics 22, no. 1 (2021): 19–34. 31107534 10.1093/biostatistics/kxz014 PMC7846150

[34] B. P. Hobbs and R. Landin , “Bayesian Basket Trial Design With Exchangeability Monitoring,” Statistics in Medicine 37, no. 25 (2018): 3557–3572. 29984488 10.1002/sim.7893

[35] ICH , “Statistical Principles for Clinical Trials,” 1998. 10.1177/030006059802600201 9602983

[36] FDA , “Adjusting for Covariates in Randomized Clinical Trials for Drugs and Biological Products. Guidance for Industry,” 2023. 10.1177/17407745251405770 41474130

[37] L. Yang and A. A. Tsiatis , “Efficiency Study of Estimators for a Treatment Effect in a Pretest–Posttest Trial,” American Statistician 55, no. 4 (2001): 314–321.

[38] A. A. Tsiatis , M. Davidian , M. Zhang , and X. Lu , “Covariate Adjustment for Two‐Sample Treatment Comparisons in Randomized Clinical Trials: A Principled Yet Flexible Approach,” Statistics in Medicine 27, no. 23 (2008): 4658–4677. 17960577 10.1002/sim.3113 PMC2562926

[39] D. A. Freedman , “On Regression Adjustments to Experimental Data,” Advances in Applied Mathematics 40 (2008): 180–193.

[40] B. Wang , E. L. Ogburn , and M. Rosenblum , “Analysis of Covariance in Randomized Trials: More Precision and Valid Confidence Intervals, Without Model Assumptions,” Biometrics 75, no. 4 (2019): 1391–1400. 31009064 10.1111/biom.13062

[41] M. M. Ananda , “Bayesian and Non‐Bayesian Solutions to Analysis of Covariance Models Under Heteroscedasticity,” Journal of Econometrics 86, no. 1 (1998): 177–192.

[42] M. Meshkani , A. Fallah , and A. Kavousi , “Bayesian Analysis of Covariance Under Inverse Gaussian Model,” Journal of Applied Statistics 43, no. 2 (2016): 280–298.

[43] H. Qi , D. Rizopoulos , and J. Van Rosmalen , “Incorporating Historical Control Information in ANCOVA Models Using the Meta‐Analytic‐Predictive Approach,” Research Synthesis Methods 13, no. 6 (2022): 681–696. 35439840 10.1002/jrsm.1561 PMC9790567

[44] H. Brückner , S. Wallot , H. Horvath , D. D. Ebert , and D. Lehr , “Effectiveness of an Online Recovery Training for Employees Exposed to Blurred Boundaries Between Work and Non‐Work: Bayesian Analysis of a Randomised Controlled Trial,” BMJ Mental Health 27, no. 1 (2024): 1–7. 10.1136/bmjment-2024-301016 PMC11033646 38642919

[45] W. Lin , “Agnostic Notes on Regression Adjustments to Experimental Data: Reexamining Freedman's Critique,” Annals of Applied Statistics 7, no. 1 (2013): 295–318.

[46] K. M. Cunanan , A. Iasonos , R. Shen , and M. Gönen , “Variance Prior Specification for a Basket Trial Design Using Bayesian Hierarchical Modeling,” Clinical Trials 16, no. 2 (2019): 142–153. 30526008 10.1177/1740774518812779 PMC6822679

[47] A. Gelman , “Prior Distributions for Variance Parameters in Hierarchical Models (Comment on Article by Browne and Draper),” Bayesian Analysis 1, no. 3 (2006): 515–534.

[48] C. N. Harrison , A. J. Mead , A. Panchal , et al., “Ruxolitinib vs Best Available Therapy for ET Intolerant or Resistant to Hydroxycarbamide,” Blood 130, no. 17 (2017): 1889–1897. 29074595 10.1182/blood-2017-05-785790 PMC6410531

[49] C. N. Harrison , J. Nangalia , R. Boucher , et al., “Ruxolitinib Versus Best Available Therapy for Polycythemia Vera Intolerant or Resistant to Hydroxycarbamide in a Randomized Trial,” Journal of Clinical Oncology 41, no. 19 (2023): 3534–3544. 37126762 10.1200/JCO.22.01935 PMC10306428

[50] A. Albert and J. A. Anderson , “On the Existence of Maximum Likelihood Estimates in Logistic Regression Models,” Biometrika 71, no. 1 (1984): 1–10.

[51] H. Zheng , M. J. Grayling , P. Mozgunov , T. Jaki , and J. M. Wason , “Bayesian Sample Size Determination in Basket Trials Borrowing Information Between Subsets,” Biostatistics 24, no. 4 (2023): 1000–1016. 35993875 10.1093/biostatistics/kxac033 PMC11616727

